# Fungal-type carbohydrate binding modules from the coccolithophore *Emiliania huxleyi* show binding affinity to cellulose and chitin

**DOI:** 10.1371/journal.pone.0197875

**Published:** 2018-05-21

**Authors:** Bart J. M. Rooijakkers, Martina S. Ikonen, Markus B. Linder

**Affiliations:** Department of Bioproducts and Biosystems, School of Chemical Engineering, Aalto University, Espoo, Finland; Institut National de la Recherche Agronomique, FRANCE

## Abstract

Six fungal-type cellulose binding domains were found in the genome of the coccolithophore *Emiliania huxleyi* and cloned and expressed in *Escherichia coli*. Sequence comparison indicate high similarity to fungal cellulose binding domains, raising the question of why these domains exist in coccolithophores. The proteins were tested for binding with cellulose and chitin as ligands, which resulted in the identification of two functional carbohydrate binding modules: EHUX2 and EHUX4. Compared to benchmark fungal cellulose binding domain Cel7A-CBM1 from *Trichoderma reesei*, these proteins showed slightly lower binding to birch and bacterial cellulose, but were more efficient chitin binders. Finally, a set of cellulose binding domains was created based on the shuffling of one well-functioning and one non-functional domain. These were characterized in order to get more information of the binding domain’s sequence–function relationship, indicating characteristic differences between the molecular basis of cellulose versus chitin recognition. As previous reports have showed the presence of cellulose in coccoliths and here we find functional cellulose binding modules, a possible connection is discussed.

## Introduction

Widespread in oceans all over the world, the marine phytoplankton called *coccolithophore*, is a type of unicellular algae from the phylum of haptophytes [1]. A number of coccolithophore species are capable of constructing *coccoliths*: exoskeletons consisting of a number of intricately formed CaCO_3_-scales that surround their cell walls. There are several indications that the coccolithophore uses so-called coccolith polysaccharides (CP) [2–4] and possibly proteins [5] for the controlled production of this interesting calcite material, although the exact form of these constituents still requires further clarification. We found a number of putative cellulose-binding protein domain sequences in the genome of the well-researched coccolithophore *Emiliania huxleyi*, and in this publication we aim to further elucidate the function of these sequences to gain insight in their possible roles for the organism.

The cellulose-binding protein domains found in cellulolytic fungal enzymes, usually in conjunction with a distinct catalytic domain, are assigned to the family 1carbohydrate-binding modules (CBM1) [6–8]. There is a large variety amongst the different CBM families in both structure and function, binding different carbohydrate molecules including xylan, cellulose, and chitin [9]. Although CBM1s are generally found in fungi, there are a number of reported cases where CBM1s are discovered in heterokonts, such as Oomycetes, brown and red algae (Rhodophyta) [10,11]. CBM1 domains have been thoroughly researched and reviewed [12,13], generally with the aim to increase knowledge regarding structure and function of cellulolytic enzymes, and to employ said enzymes in the lignocellulose industry and biofuel production [14,15]. CBM1s are well-defined polypeptides consisting of typically 36 amino acids (~3.8 kDa), held together in a compact pyramid-like structure by two highly conserved disulfide bridges. One face of the pyramid interacts with the surface microcrystalline cellulose, where three aromatic residues (most often Trp or Tyr) facilitate specific cellulose binding affinity by π-stacking, in addition to hydrogen bonding [16–19].

Coccolithophores are the ocean’s main calcifiers, and intensely studied for their influence on the global carbon cycle and climate change. Even so, many questions still surround their coccoliths. Firstly, it is unknown why exactly coccolithophores create their exoskeletons. It has been suggested that the coccoliths regulate cell sinking, or protect against UV or light [3,20]. Secondly, only a number of species are coccolith-covered and even the ones that can produce calcite scales, exist in forms with and without scales [3,21]. Finding answers to these questions is challenging due to the high level of variety that exists between and even within haptophyte taxa [22].

One aspect that has been extensively reviewed is the coccolith composition and formation, although questions remain. The elaborate calcite-based scales are produced one-by-one through biomineralization intracellularly after which the disks are transferred out of the cell. Using CO_2_ or bicarbonate ions from the ocean as carbon source, the calcification process occurs on organic baseplates containing polysaccharides and probably proteins inside dedicated intracellular vesicles cells [3,5]. The suggested role for these polysaccharides is for precise control of calcite crystallization: they form an inner lining of the coccolith vesicle and seem to have a key role in giving the coccolith its characteristic shape, through strong anionic binding to calcium [2,23,24]. Interestingly, it has been shown experimentally that cellulose is present in coccoliths [25–27], although its precise function is unknown. Also, early reports suggested the presence of a ‘cellulosic glycoprotein’ with protein covalently linked to cellulose [25].

No proof of chitin in coccoliths has been presented as of yet, even though chitin is often encountered as a scaffold in calcite-based biomineralization–especially in marine organisms such as mollusk’s shells [28]. The way chitin is usually involved in the formation of CaCO_3_ materials is by forming a matrix with super-saturated amorphous calcium carbonate (ACC) and hydrophobic silk-like proteins, which functions as a controlled micro-environment for calcite crystal growth. However, there is currently no evidence of ACC existing in coccolithophores.

The most abundant and widespread coccolithophore species is *E*. *huxleyi*, and its entire pan-genome has recently been sequenced [29]. In the now available genome data, a number of cellulose-binding protein sequences were found, in addition to several chitin-binding domains. Since these fungal-type CBM1 appear to be present unexpectedly in coccolithophores, and polysaccharides as well as proteins are anticipated to play a directing role in the calcification process, we hypothesize that the identified CBM1s might be structural proteins involved cellulose interactions, possibly in processes targeted to the coccolith. There are no direct indications for this hypothesis, and to investigate the functioning and possible role of these CBM1s from *E*. *huxleyi*, they have been cloned and produced in *E*. *coli* and their binding to several cellulose and chitin ligands was studied for evaluating possible functional roles.

## Results

### Construct design and protein production

**Fig 1:**
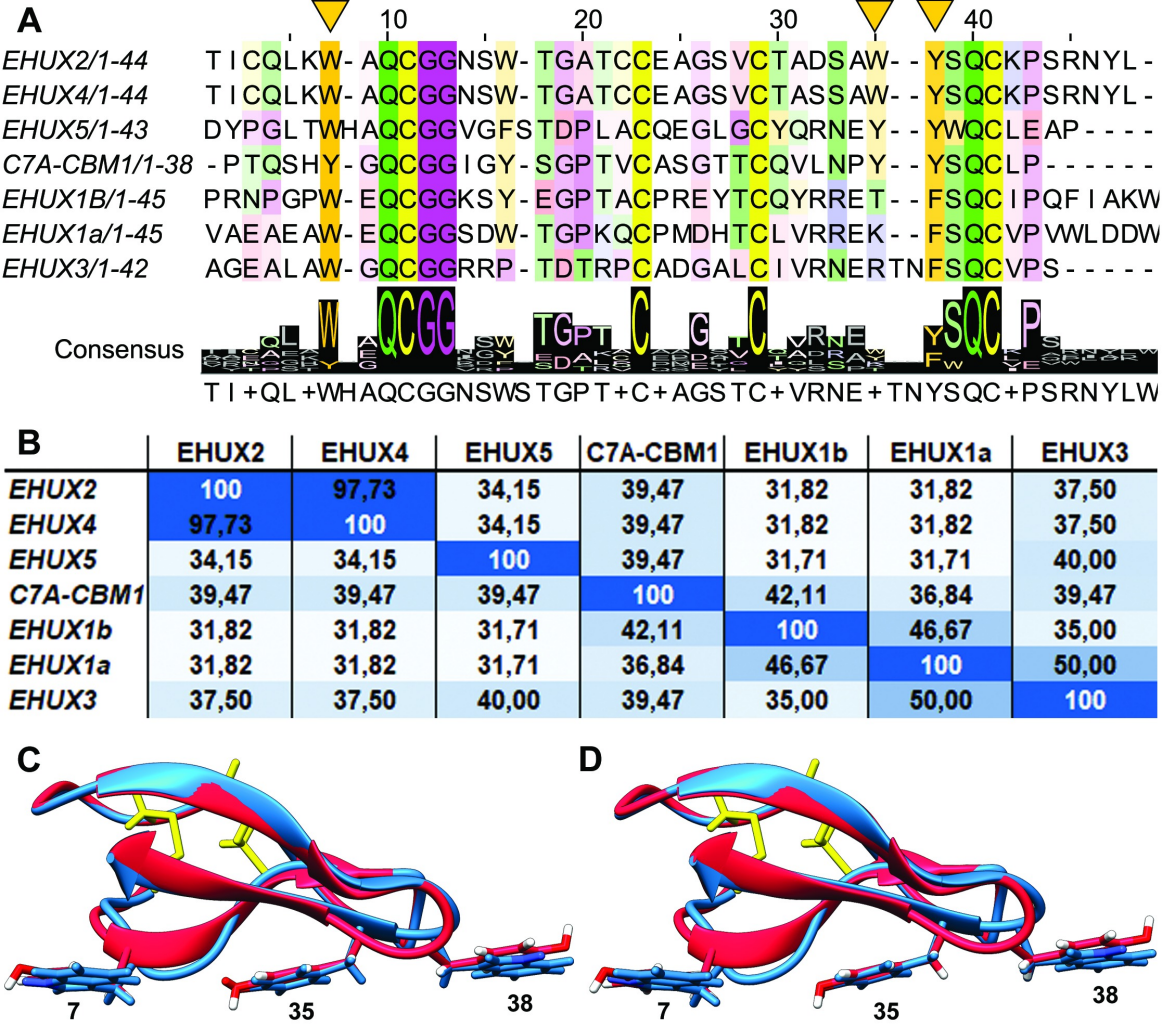
**(A)** Multiple sequence alignment of CBM1 domains found in *E*. huxleyi, with benchmark CBM1 from Cel7A from *T*. *reesei* in the middle. The consensus sequence is visualized underneath the MSA. Residues are colored according to Zappo color scheme, with conserved residues colored more intensely. The three key aromatic residues involved in cellulose binding (position 7, 35, 38) are indicated with yellow triangles above the sequences. Alignment made with MUSCLE and formatted with Jalview 2 [32,33]. **(B)** Distance identity matrix of CBM1 domains found in *E*. *huxleyi* and benchmark CBM1 from Cel7A, displaying pairwise sequence identities in percentages (X of Y positions identical). The intensity of the blue color correlates with increasing sequence similarity. **(C-D)** Homology models of the (**C**) EHUX2 and (**D**) EHUX4 CBM1 domains, displayed as blue ribbons, with Cel7A-CBM1 as overlay, displayed in red. Cysteine bridges and the side-chains of residues that are important for binding are shown. Homology models were created by SWISS-MODEL [34] with the crystal structure of Cel7A-CBM1 (PDB ID: 1CBH [16]) as a template.

The *E*. *huxleyi* genome [29] was screened using Pfam (ID: PF00734) [30] and BLAST (with the Cel7A-CBM1 sequence) and five proteins were found containing a total of six probable CBM1 domains. A multiple sequence alignment (MSA) as well as an identity distance matrix can be seen in Fig 1, which shows the homology of these CBM1s to each other as well as to the Cel7A-CBM1. Pairwise sequence identities between the CBM1 homologs range between 31.71% (14 out of 45 residues identical) up to 97.73% (44 out of 45 residues identical), in the case of EHUX2 and EHUX4. Cel7A (previously cellobiohydrolase 1, CBH1) is a well-characterized cellulase from the fungus *T*. *reesei* [12,31] and since there is extensive binding data published regarding its CBM1 domain, it was chosen to be used as a reference CBM1 in this study. In the pairwise comparison, it showed intermediate values for sequence identity in comparison to the group of EHUX CBMs. Structures and homology models are shown in S1 and S2 Figs.

The five genes that the identified CBM1 domains were found in, contain a variety of other functionalities. From sequence predictions, all proteins are expected to be hydrolyzing O-glycosyl compounds and anchored in the membrane extracellularly. The protein that contains EHUX1a and EHUX1b also shows a highly Asp-rich domain, whereas EHUX2 is predicted to be a tyrosine kinase. In S1 Table the sequence identifiers (GI), names and probable functions of the CBM1-containing proteins from *E*. *huxleyi* can be seen. The associated functions were obtained with Gene Ontology (GO) and Interpro sequence-based predictions.

To investigate the functioning of the proteins with CBM1 domains found in the genome of *E*. *huxleyi*, constructs were designed containing an N-terminal pelB signal peptide, Alkaline Phosphatase (AP) as a carrier protein and the CBM1 of interest with a His-tag on the C-terminus (S3 Fig). The AP was chosen for previous good expression results and because it can be visualized using a colorimetric assay. The proteins were cloned and expressed in *Escherichia coli* with a second CyDisCo plasmid, carrying the genetic code for two proteins that actively aid in the proper formation of the disulfide bridges in the cytoplasm [35,36], reaching expression levels of 100–300 mg protein / L culture.

**Fig 2:**
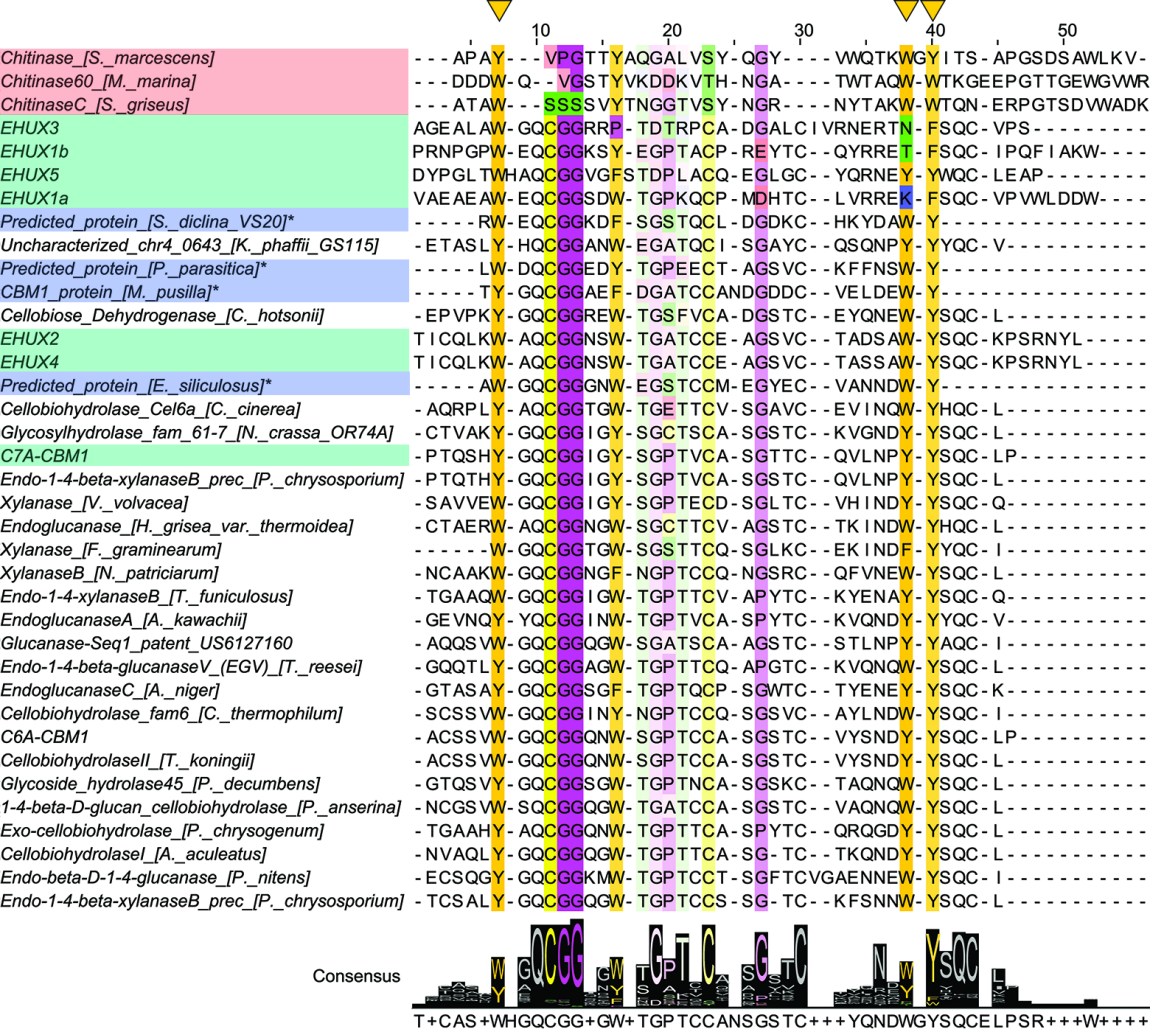
Multiple sequence alignment of the investigated *E*. *huxleyi* CBM1 domains and selected related sequences. In this MSA, the six *E*. *huxleyi* CBM1 domains are shown together with predicted non-fungal CBM1 domains, a selection of biochemically characterized CBMs from the CAZy database: CBM5 and fungal CBM1. The consensus sequence is visualized underneath the MSA. Residues are colored according to Zappo color scheme, with conserved residues colored more intensely. The three key aromatic residues involved in cellulose binding (position 7, 35, 38) are indicated with yellow triangles above the sequences. Alignment made with MUSCLE and formatted with Jalview 2 [32,33].

A sequence alignment (Fig 2) with a dataset consisting of the six CBM1 domains found in *E*. *huxleyi*, Cel7A-CBM1, different biochemically characterized CBMs selected from the CAZy database [37,38] and predicted proteins from the Pfam database [30] show how conserved amino acids are grouped within the sequences. The evolutionary relation between sequences could not be reliably determined since phylogenetic trees constructed gave bootstrap values below 20%. The comparison in Fig 2 shows that only very short stretches of the sequences are variable, and do not allow reliable phylogenetic analysis.

### Binding assays

The purified proteins were tested in binding studies, with three polysaccharides as ligand: *CNF (Cellulose Nano Fibrils)*, *Bacterial Cellulose (BC)* and *Chitin Nano Crystals (ChNC)*. A starting protein concentration ranging from 1–5 μM was incubated with a known amount of ligand and subsequently separated by centrifugation, after which the resulting protein amounts were measured on reverse-phase UHPLC (Ultra High Performance Liquid Chromatography) and compared to a control without ligand.

**Fig 3:**
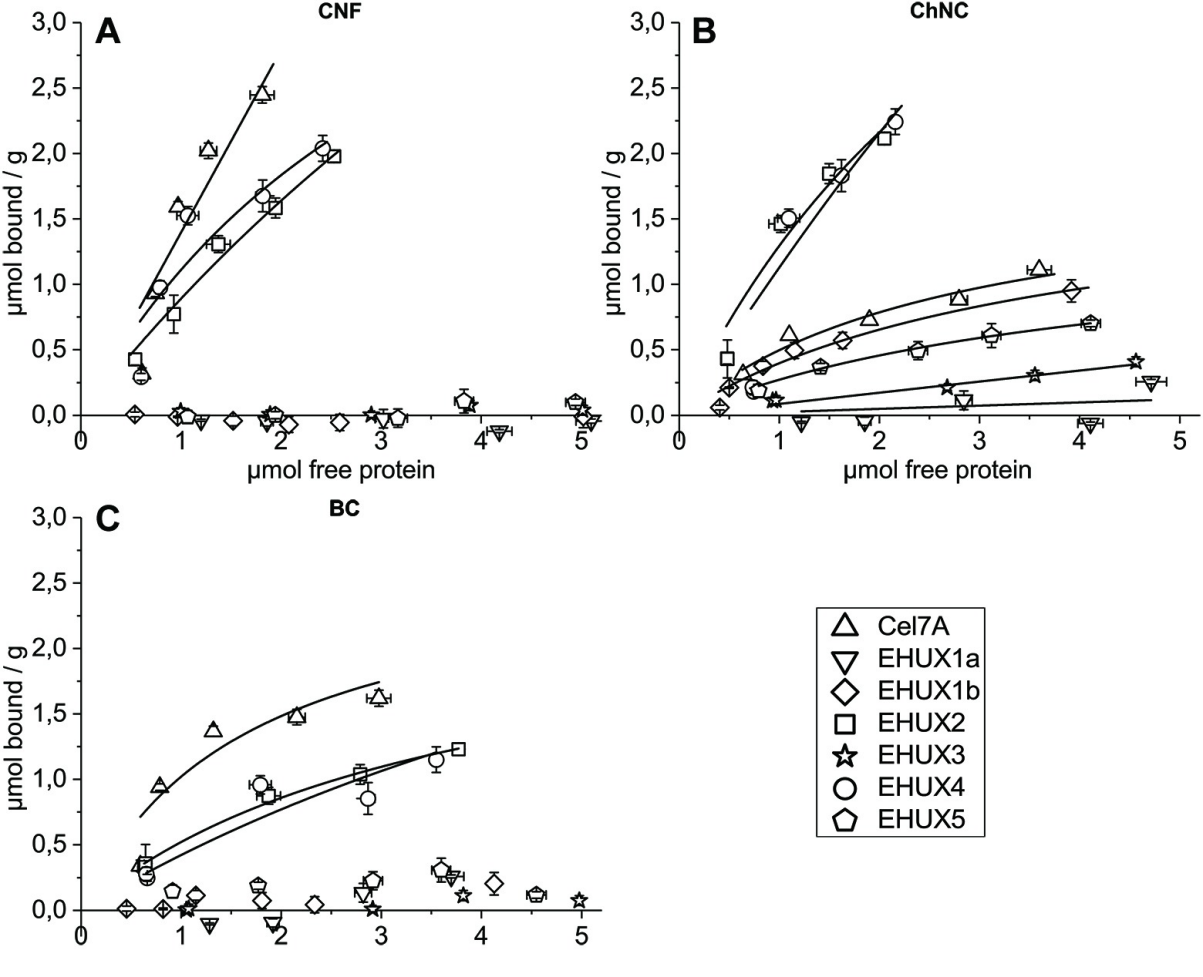
**Binding isotherms of all measured CBM1 proteins** (**A**) with cellulose nanofibrils (CNF), (**B**) with chitin nanocrystals (ChNC) and (**C**) with bacterial cellulose (BC) as a ligand, displayed as μmol of bound AP-CBM1 protein / gram of ligand, against μmol of free protein.

In Fig 3 the resulting binding isotherms of all tested *E*. *huxleyi* proteins can be found, in addition to the benchmark protein Cel7A-CBM1, for three different ligands. The graphs show that two of the proteins from *E*. *huxleyi* are actually efficient cellulose binders, namely EHUX2 and EHUX4. Both proteins bind to the CNF ligand as well as to BC, both at slightly lower levels than the benchmark protein Cel7A-CBM1. Cel7A-CBM1 still binds most effectively to both cellulose ligands, and all three binding CBM1s have shown more efficient binding to CNF in contrast to BC, which is in line with previous reports [39]. The other CBM1 proteins from *E*. *huxleyi* show little to no binding to either cellulose ligand.

In contrast, the binding isotherm for ChNC shows that all CBM1s bind the ligand to some extent, effectively spreading data points across the graph. EHUX2 and EHUX4 show a clearly better binding (up to 2.2 μmol/g) than the other proteins, binding to a similar extent as in the case of CNF. Notably, Cel7A-CBM1 binds significantly less effectively to chitin than to cellulose.

**Fig 4:**
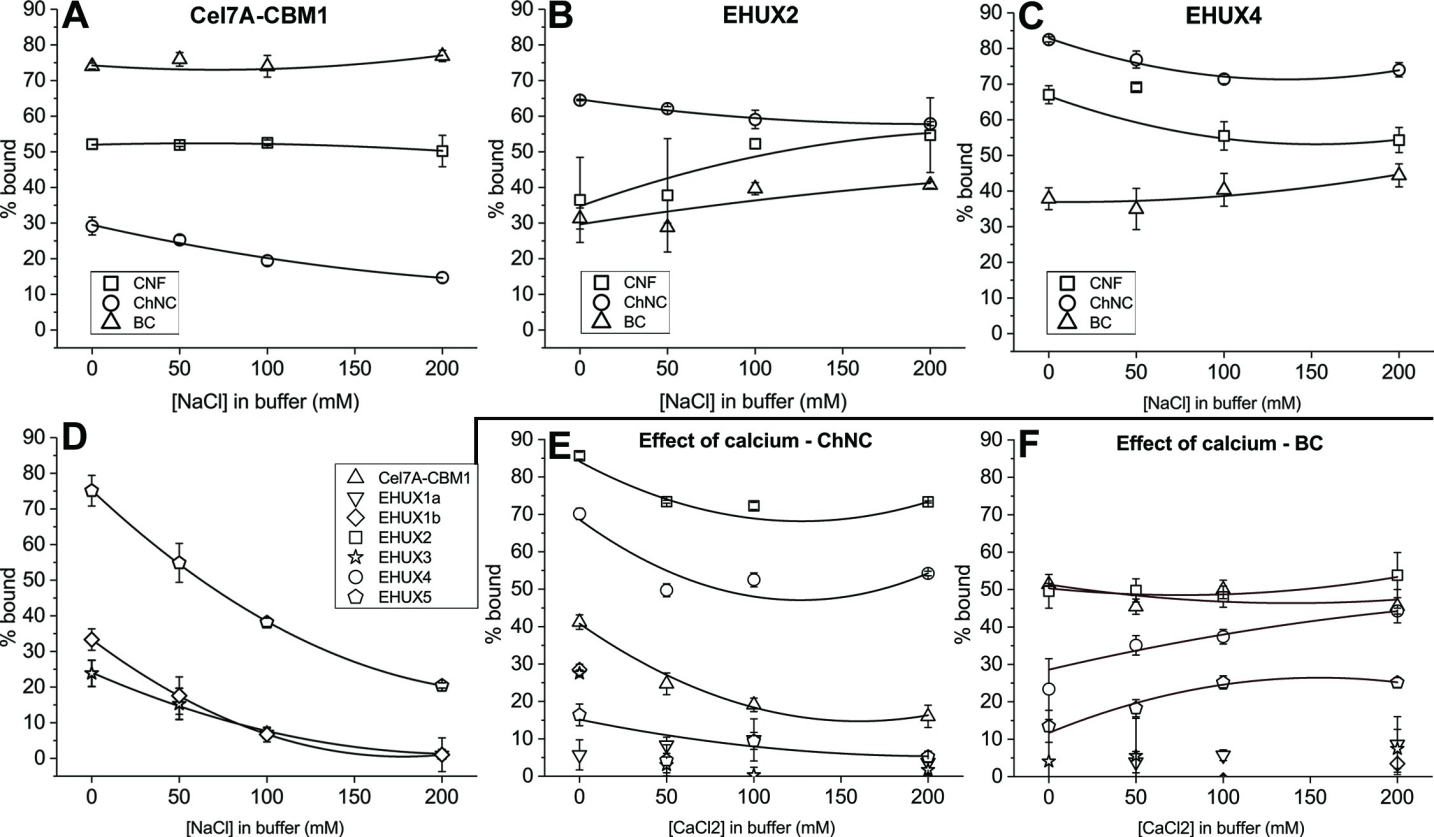
Binding of CBM1s in buffers with NaCl and CaCl_2_. The effect of increasing ionic strength as well as calcium concentration was studied in detail for all proteins that showed binding to the ligands. The binding is displayed in percentage of AP-CBM1 protein bound / gram of carbohydrate ligand, in buffers containing increasing concentrations of NaCl or CaCl_2_. The CBM1s that show binding are accompanied by viewing guide lines. (**A**) Cel7A-CBM1 (**B**) EHUX2 (**C**) EHUX4(**D**) EHUX1b, EHUX3, EHUX5 with ChNC as binding ligand. Calcium concentration effect on binding to ChNC (**E**) and BC (**F**) for all proteins.

Specific CBM1-cellulose binding is considered to be facilitated by π-π stacking of a number of key aromatic residues that can be found on position 7, 35 and 38 in the alignment in Fig 1, as well as hydrogen bonding. For this reason, the effect of increasing ionic strength was studied, in order to assess the contribution of non-specific, possibly electrostatic interactions to binding. A number of data points were collected of all tested proteins that showed binding, at an initial protein concentration in the lower-mid range of the binding curve (2.5 μM) and in buffers with increasing salt concentrations: 0, 50, 100 and 200 mM NaCl. The results are shown in Fig 4, in percentage of CBM1 bound. For the proteins with binding affinity for CNF, binding slightly declines with increased salt amounts, but the majority of the binding seems to come from specific structure-related binding. All proteins except EHUX2, EHUX4 and Cel7A-CBM1, show a large decrease in binding to chitin as salt concentration increases, even as initial binding is significant. This suggests that there is a significant component of electrostatic interactions in the binding, indicating that it may be non-specific. In contrast, the binding of EHUX2 and EHUX4 to chitin is only slightly affected by increasing salt concentrations, supporting the conclusion that the binding is specific, considering the fact that binding of CBM1 has been shown to consist of mostly pi-stacking and hydrogen bonding.

Similarly, the effect of calcium concentration on the binding of the CBM1s was investigated, as the calcium is available in and around calcite-creating coccolithophores, and some CBM families other than CBM1 require calcium ions for functioning. The binding of all CBM1 proteins to chitin and cellulose were tested in four different calcium concentrations: 0, 50, 100, 200 mM CaCl_2_ (Fig 4, panels E and F). Just as in the salt binding assays, an increase in CaCl_2_ salt shows a decrease in binding to chitin but not cellulose, confirming that part of the binding consists of electrostatic interactions. The same proteins that show binding functionality (Cel7A-CBM1, EHUX2 and EHUX4) show similar binding levels here. As in the binding isotherm data (Fig 3C), EHUX5 showed a slightly higher binding to BC than EHUX1a, EHUX1b, and EHUX3. CaCl_2_ affected EHUX4 binding to BC slightly up to 100mM, and more clearly at the high concentration of 200 mM CaCl_2_.

**Fig 5:**
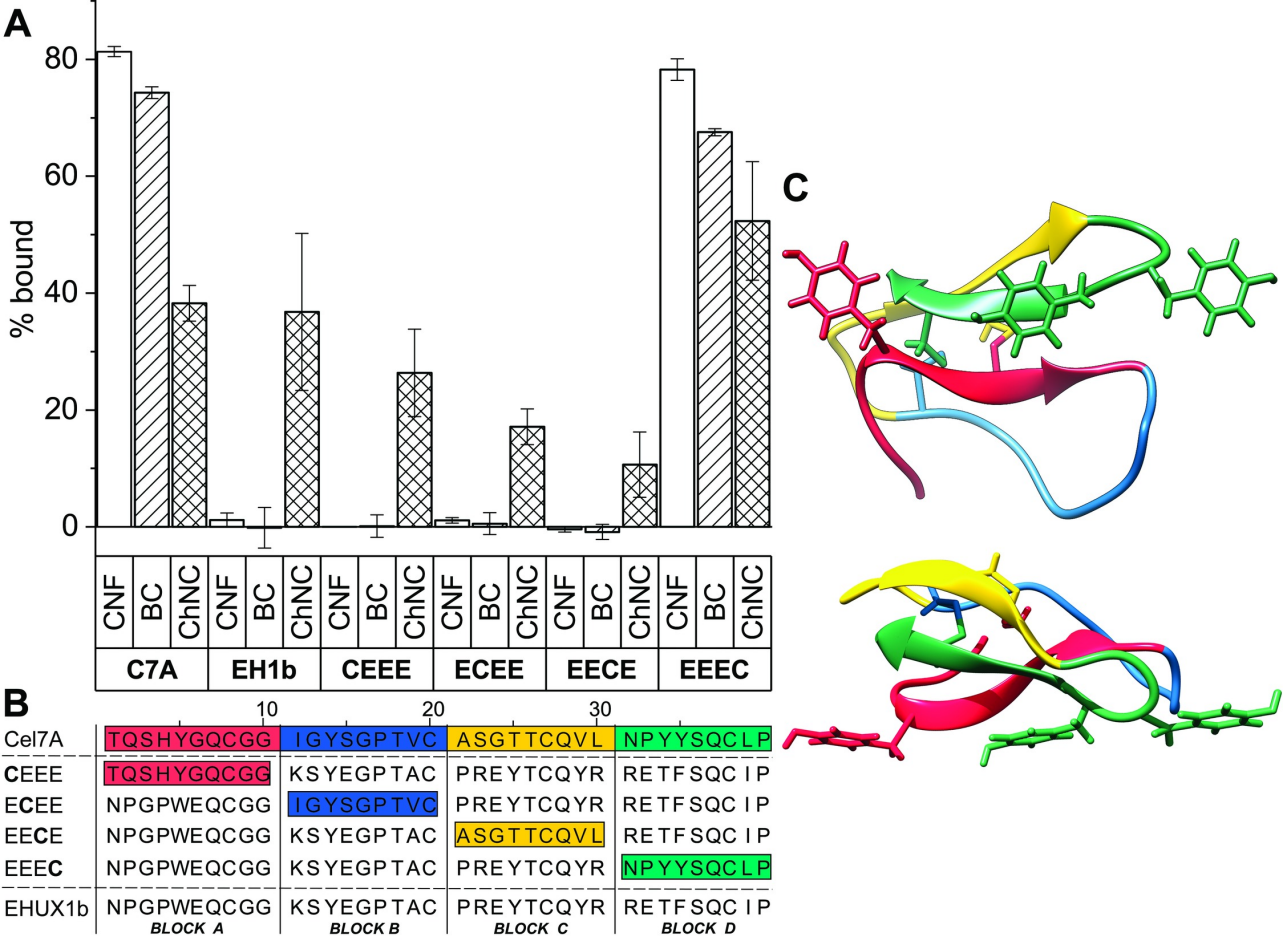
Cel7A-EHUX1b segment shuffle experiment. **(A)** Bar graph of the binding of Cel7A-CBM1, EHUX1b and 4 shuffled proteins, with the percentage of the protein bound to CNF, BC and ChNC shown on the y-axis. The shuffled proteins are labeled with four letter codes, where each of the four fragments is represented by the starting letter of the CBM1 they originate from (C = Cel7A; E = EHUX1b). (**B**) Sequences of Cel7A-CBM1, EHUX1b and the shuffled proteins. The four segments from Cel7A-CBM1 are color coded, corresponding with the 3D structure of Cel7A-CBM1 shown in (**C**). (**C**) Cel7A-CBM1 crystal structures with the segments use to create the shuffled proteins in four different colors. 3D structure shown as cartoon, with a view on the binding face as well as a front-side view. Image created with Chimera [40], PDB ID: 1CBH [16].

To further study the relation between the intermediate binding to chitin and very low binding to cellulose shown by many of the EHUX CBMs (Fig 3), we investigated the EHUX1b sequence in more detail. The sequence of EHUX1b was divided into four parts and each of them were in turn exchanged with the corresponding sequence of the extensively characterized benchmark Cel7A-CBM1 (Fig 4). The resulting four sequences were cloned and expressed in *E*. *coli*, and compared to both Cel7A-CBM1 and EHUX1b for binding. The reshuffled EHUX1b-Cel7A proteins are here referred to with four letter codes (sequences can be found in Fig 5), where each of the four fragments is represented by the starting letter of the CBM1 they originate from. The three combinations CEEE, ECEE, and EECE show a decreased binding to chitin, but still with the general observation of intermediate chitin binding and no cellulose binding. The EEEC combination, however, shows a cellulose- and chitin-binding capability comparable to that of Cel7A-CBM1. The shuffling experiment shows that grafting only the last 1/4^th^ and in practice only 5 residues of the Cel7A into the EHUX1b, can make a very similar protein to Cel7A. Also, the results of this binding assay (Fig 5), also shows similar binding levels as measured with UPLC for Cel7A-CBM1 and EHUX1b as with the colorimetric AP-assay. In the AP-assay 1% of BSA was added, showing that AP does not contribute to non-specific binding.

## Discussion

The investigation of the binding capabilities of the CBM1s from *E*. *huxleyi* clearly identified two cellulose-binding proteins: EHUX2 and EHUX4. Compared to the benchmark protein Cel7A-CBM1, they bind less effectively to both cellulose ligands, but EHUX2 and EHUX4 are significantly better ChNC binders than Cel7A-CBM1, and they bind chitin more effectively than either cellulose ligand (Fig 3). Not much is known regarding CBM1 domains binding to chitin, but fungal-type binding domains with higher binding efficiency for chitin than for cellulose has been demonstrated before [41]. The fact that CBM1s show more binding affinity for nanofibrils instead of crystalline bacterial cellulose, has been reported previously [39], and could be attributed to the higher surface area of CNF per weight, as well as the additional binding mode that Cel7A-CBM1 seems to have on CNF but not on BC. As a comparison to previously published work we note that the Cel7A-CBM1 construct used here showed the same binding affinity for cellulose, with a partitioning coefficient of 1.4 L/g, as reported for the Cel7A CBM previously [39,42].

Where all other CBM1 proteins clearly show no binding to BC and CNF, the binding isotherm for chitin (Fig 3B) shows some level of binding affinity for all proteins. The maximum binding levels are spread between around 0 and 1 μmol CBM1 / g ChNC, with the exception of functional binders EHUX2 and EHUX4, which stand out above the rest with up to 2.2 μmol of bound CBM1 per gram of ChNC. This indicates that the CBM1s EHUX1b, EHUX3 and EHUX5 seem to have a lower binding specificity for chitin than for cellulose, perhaps due to non-specific electrostatic interactions contributing more to the binding.

Additional binding studies using buffers with increasing salt concentrations show that for the domains that bind to cellulosic ligands, binding either stays constant or is reduced only slightly at higher salt concentrations. Some increase in binding could be observed with for example CaCl_2_ for EHUX4 and EHUX5 but only at high concentration, indicating a salting-in effect rather than indicating calcium dependency. As the presence of salt will screen electrostatic interactions, this indicates that the contribution of those contacts is only minor, and that the majority of cellulose binding results from the specific functioning of the CBM1 binding face. However, for all the CBM1 proteins that bind only to chitin, higher NaCl concentrations had a clear diminishing effect on binding with a similar but less pronounced effect also for CaCl_2_. It is interesting to see that the contribution of screenable electrostatic binding seems to be much higher in the case of chitin, which corresponds with the ChNC binding isotherm that shows that almost every investigated CBM1 has at least some level of binding to chitin, suggesting binding through other ways than the specific, conserved binding residues. The cellulose-binding domains EHUX2 and EHUX4 retain a significant portion of binding (60% and 75% of CBM1 bound, respectively) even in the highest salt concentration (both NaCl and CaCl_2_), which once again depicts these domains from *E*. *huxleyi* as effective binders to both chitin and cellulose.

When the sequences of the CBM1s are analyzed more in depth, aided by the multiple sequence alignment shown in Fig 1, a few observations can be made: Firstly, the three aromatic residues that are located in the binding face of the domain (Tyr7, Tyr35, Tyr38 in Cel7A-CBM1) are highly conserved throughout CBM1 domains and have been proven to be crucial in the binding to polysaccharides, at least in the case of Cel7A-CBM1. [18,43] Also, these aromatic residues are followed by highly conserved segments with cysteines (Gln-Cys-Gly-Gly and Ser-Gln-Cys), which are expected to contribute to the specific, compact pyramid structure of the CBM1 domain, held together by two disulfide bridges.

There are a number of differences between the CBM1s of *E*. *huxleyi* and Cel7A, such as the additional pair of cysteines in EHUX2 and EHUX4, which are positioned next to each other when the predicted 3D structure is taken into consideration (S2 Fig). In addition, four out of five sequences have one or two additional Tyr or Phe just after the CBM1 domain, and all EHUX CBM1s contain numerous charged residues, whereas Cel7A-CBM1 is without charge.

EHUX1a, EHUX1b, EHUX3 show no binding to cellulose and lack aromatic residues in position 35. Previously it has been shown for Cel7A-CBM1 that affinity towards cellulose is lost if this residue is mutated to a non-aromatic one [18], which fits well with the observed lack of binding of these three *E*. *huxleyi* proteins. However, EHUX5 does contain a Tyr in position 35, but still shows only weak binding. There is no obvious reason for the lack of binding but we note an unusual Trp in position 39 and a His insertion in position 8 that both may cause structural perturbations, possibly leading to loss of binding. These same proteins (specifically EHUX1a and EHUX1b) also have a number of positively charged residues just in front of the final two binding aromatic residues, which could interfere with the carbohydrate binding.

By reshuffling the sequences of Cel7A-CBM1 and EHUX1b-CBM1 we could obtain more insight into sequence-function relationship of the CBM1 domain. Where three out of four reshuffled EHUX1b-Cel7A proteins (CEEE, ECEE, EECE) act in line with EHUX1b and do not bind to CNF, the EEEC reshuffled protein regains the full binding capability of Cel7A-CBM1 for all three ligands, and binds chitin even better. A look at the sequence of the CBM1s (Fig 1) learns that the double tyrosine residues in Cel7A-CBM1 in place of the threonine and phenylalanine in EHUX1b-CBM1 seem to be key in binding to cellulose and–to a lesser extent–chitin. The regaining of binding in this shuffling experiment confirm that the CBM scaffold can easily be modified for function by the change of only a few amino acids and that the other differences in EHUX1b compared to Cel7a-CBM1 do not make the scaffold itself non-functional. The CBM1-scaffold itself seems to be very robust.

This result corroborates our understanding that the original function of CBM1 is to bind cellulosic ligands, since the change of a small part of the domain can completely remove its binding capabilities. Vice versa, altering just a few residues of a non-functional binding domain can result in that protein regaining its cellulose or chitin binding function. This experiment shows the unexpected result that cellulose binding function is more critically dependent on structure than the chitin affinity, since cellulose affinity is completely lost with the altering of one segment of the domain, whereas chitin binding functionality seems less specific and is always present to some extent.

The fact that five genomic sequences encoding homologs of fungal cellulose-binding domains were found in the genome of *E*. *huxleyi* is intriguing. The pfam database that contains all CBM1 domains–predicted based on sequence–displays that of the 2702 unique sequences containing a CBM1, 2523 come from fungi. Other eukaryote genomes that have CBM1-containing sequences include green algae, stramenophiles (algae, diatoms) and Haptophyceae (7, mostly *E*. *huxleyi*). Coccolithophores are a type of eukaryotic algae and part of the division of Haptophyta; although their taxonomy is an ongoing topic of discussion, which illustrates their peculiar isolated position in the eukaryotic tree of life. [1,22,44] The distribution of CBM1 over organisms shows that it is indeed found mostly in fungi, but also in some unicellular marine organisms, indicating that at one point in history, the genetic information of CBM1 domains went from fungi to eukaryote unicellular marine organisms, or vice versa. The fact that the CBM1 domain is only around 30 amino acids long and often part of a protein many times that length, makes a proper phylogenetic analysis unfeasible, yielding unreliable phylogenetic trees with very low bootstrap values. Of the CBM1 sequence only very short stretches are variable and not directly involved in either the function of the protein or have a structural role for the scaffold (Fig 2). Residues before 7 and after 43 are parts of linkers or variable termini with unclear origin. Only a short continuous stretch between the Cys at position 30 and the aromatic reside at position 38 seems to be variable and not conserved for function or being critical for structure. This also explains the variety amongst the CBM1 sequences from *E*. *huxleyi* and why it is hard to pinpoint how and where in the evolutionary history coccolithophores obtained the genetic data for CBM1 domains. Additional full genome-sequencing of related organisms could shed light on this question. Meanwhile, functional studies such as presented here help to gain understanding.

A closer look at the full sequences of the CBM1-containing proteins from *E*. *huxleyi* (S1 Table, shows that they are all predicted to have several transmembrane (TM) regions, and all proteins are likely to be on the outside of the plasma membrane. None of the proteins are related to carbohydrate metabolism other than their CBM1 domains and sources of cellulose in oceans (from algae, tunicates) are limited. This promotes the possibility that some of these CBM1-containing proteins are either genetic remnants of an evolutionary past, or have evolved to gain a different function: as part of protein signaling chains or targeting of proteins to cellulose and chitin molecules, or perhaps that they are involved in extracellular cellulose scaffolding for coccoliths.

The fact that this coccolithophore contains two functional CBM1s that bind both cellulose and chitin, raises the question of the function of these carbohydrates in the organism. Both proteins containing these binding domains are predicted to be extracellular. Related organisms such as green algae are capable of synthesizing cellulose [45], and chitin is often found as part of the mineralized biocomposite material in the shells of marine organisms. As polysaccharides are known elements of coccolith formation, it is possible that *E*. *huxleyi* produces its own cellulose for structural purposes, even though no known cellulose synthase homolog sequences were found in its genome. [29] Since previous research has implied the presence of cellulose and proteins in the exoskeletons surrounding these organisms, the finding of cellulose-binding proteins in *E*. *huxleyi* could signify a role for cellulose as scaffold in coccoliths, or a role as structural proteins for the CBM1s.

## Materials and methods

### Plasmid preparation

The CBM1 constructs were designed to have a carrier protein fused to the CBM1 domain by a flexible linker, in addition to the N-terminal pelB signal peptide and a 6His purification tag on the C-terminus. Alkaline Phosphatase (AP) was chosen as carrier protein since it was known to produce well and could be used in colorimetric assays. The pelB, linker, and CBM1 sequences were ordered as GeneArt Strings (ThermoFisher) and the AP segment was obtained with PCR (using KAPA HiFi DNA Polymerase from KAPA Biosystems and primers from Eurofins), all flanked with BsaI restriction sites and appropriate 4bp overlaps. Using the Golden Gate cloning method [46] the pieces were combined (digest with BsaI-HF from NEB) and ligated (T4 DNA Ligase NEB) into a pET28a (+) expression vector and transformed in chemically competent TOP10 *E*. *coli* cells. The resulting plasmids were obtained by miniprep (NucleoSpin Plasmid from Macherey-Nagel). The resulting constructs were verified by 1% agarose gel electrophoresis and sequencing (FIMM, Helsinki).

### Protein expression and purification

The CBM1 constructs were co-transformed into chemically competent BL21(DE3) *E*. *coli* cells with CyDisCo plasmid pMJS205 [35] (which was employed to ensure the formation of disulfide bridges in the CBM1 domain and AP). One colony was picked and used to grow a pre-culture in LB medium supplemented with kanamycin (50 mg/L) and chloramphenicol (35 mg/L), for 6–8 h at 37°C with vigorous shaking. 500 mL of EnpressoB growth system (BioSilta), was supplemented with kanamycin (50 mg/L) and chloramphenicol (35 mg/L) and inoculated with 1:25 of the pre-culture, and grown for 15–18 h at 30°C, 230 rpm. Expression was induced with IPTG (final concentration 500μM), incubating the culture another 24 h at 30°C, 230 rpm.

The cells were then harvested by centrifugation (24 471 x *g* (rotor radius 15.2 cm), 4°C, 30 min), removal of the media and resuspended in lysis buffer (20 mM NaH_2_PO_4_, 20 mM imidazole, 500 mM NaCl, 1mg/mL fresh lysozyme, 10 μg/mL DNAse I, 10 μg/mL MgCl_2_, protease inhibitor cocktail (Sigma Aldrich)) at 4°C. After 30 min shaking at 4°C, the cell suspension was further lysed by running it 2–3 times through an EmulsiFlex-C3 homogenizer (Avestin, Inc.), after which the cell debris was removed by centrifugation (24 471 x *g* (rotor radius 15.2 cm), 4°C, 30 min).

The proteins were purified from the resulting lysate using GE healthcare ÄKTA Pure LC system with HisTrap IMAC columns. The CBMs were verified by SDS-PAGE analysis with Coomassie Blue staining. Protein concentrations were determined by measuring the absorption at 280 nm using a Varian Cary 50 UV-VIS spectrophotometer, corrected for background and calculated using the extinction coefficient, predicted based on protein sequence by ProtParam [47].

### Binding ligands

Cellulose nanofibrils (CNF) were prepared by fluidizing never-dried bleached birch pulp in deionized water six times (six passes) using a Voith LR40 homogenizer at 300kWh/t net specific energy with specific edge load of 0.5 J/m and refiner speed of 150. The resulting samples with a solid content of 2% w/v were then used to make a 1 mg/mL solution in water with 0.02% NaN_3_, stored at 4°C and taken out to room temperature 1 hour prior to use.

Bacterial cellulose (BC) was prepared from cellulose mats obtained from *Acetobacter xylinum* cultures. The mats were first cut to smaller pieces and blended for 15 min with household blender in deionized water to obtain a concentration of 0.5% w/v, followed by 10 min homogenization using a Polytron PT 3100 D system. Finally the samples were homogenized six times using the Voith LR40 homogenizer with similar parameters as for plant cellulose. A similar 1 mg/mL solution was made of BC in water with 0.02% NaN_3_, stored at 4°C and taken out to room temperature 1 hour prior to use.

Chitin nanocrystals (ChNC) were prepared from purified chitin flakes from shrimp shells (Sigma Aldrich) by a 90 min hydrolysis with 3M HCl at 90°C. Subsequently, the acid residue was washed out and the ChNC were further purified by dialysis for 2 days, after which the pH of the suspension adjusted to pH4 with 1M HCl. The resulting ChNC dispersion with a solid content of 2% w/v was tip sonicated (for 2 min with pulses of 2 sec on and 2 sec off at 20% amplitude) and used to make a 1 mg/mL solution in MQ with 0.02% NaN_3_ and stored at 4°C. The degree of acetylation of the ChNC was calculated from ^13^C NMR-spectra to be 99.6%.

### Binding assay

The carbohydrate binding assays were performed by mixing 100μL CBM1 protein (50mM TrisHCl pH 7 buffer) with both 100μL MQ (control) and 100μL 1 mg/mL binding ligand (CNF, BC or ChNC) to a final protein concentration of 1, 2, 3, 4 or 5 μM, followed by incubation at 23°C for 1 h. The dispersion is then centrifuged at 23°C for 2 min at 21 130 x *g* (rotor radius 8.4 cm) to separate the supernatant (containing the non-bound CBM1 protein) for analysis by SDS-PAGE or UHPLC. A Vanquish UHPLC (Thermo Scientific) was used to analyze and quantify the non-bound proteins, by injecting 20 μL of the supernatant on a BioBasic 4 column and eluting by a gradient from water to 0–60% acetonitrile, both containing 0.1% trifluoroacetic acid. For both the NaCl (salt assay) and CaCl_2_ (calcium assay) binding experiments were performed as described above, for one initial protein concentration in the middle of the binding curve (2.5 μM) and in four buffers (same as above 50mM TrisHCl pH 7) substituted with the increasing amounts of salt.

### Phylogenetic analysis

The *E*. *huxleyi* genome [29] was screened by a BLAST [48] search based on the *Trichoderma reesei* Cel7A CBM1 sequence and by searching for putative proteins with the Pfam ID for CBM1 domains: PF00734 [30]. The genome was further examined for CBM1 homologs by looking at Gene Ontology, InterPro and KEGG Metabolic Pathways. A MUSCLE [32] multiple sequence alignment was performed (default parameters) to compare all CBM1-containing sequences from *E*. *huxleyi* with Cel7A-CBM1 and visualized with Jalview [33]. The pairwise sequence identities were taken from this same MUSCLE MSA and visualized in a heatmap distance identity matrix. A dataset was created with the six *E*. *huxleyi* CBM1 domains, Cel7A-CBM1 and a randomly selected mix of verified CBM1-containing proteins from Carbohydrate-Active Enzymes (CAZy) database [37,38] and a few predicted CBM1 proteins from the Pfam database [30]. A MUSCLE MSA was created with this dataset (default parameters).

### Alkaline phosphatase assay (AP-assay)

The colorimetric AP-assay was used to measure the concentrations of the AP-CBM1 proteins directly from lysate, which in turn could be used to ascertain their binding. The concentration of protein in lysate was determined by measuring their AP activity and comparing that to the AP activity of pure AP-CBM1 protein. Then, in 200 μL total volume, 40 or 80 μg of the ligand (CNF, BC or ChNC) or an equal volume of MQ (control sample) was mixed in a tube containing 0.3 μM AP-CBM1 protein, 10 mg/mL BSA (Sigma-Aldrich A2153), buffered in 50 mM sodium acetate, pH 5. The tubes were mixed well and incubated at 23°C for 1h. The samples were then centrifuged at 23°C for 2 min at 21 130 x *g* (rotor radius 8.4 cm), and the supernatant was analyzed with SDS-PAGE or AP activity assay, as described below.

Samples of 50 μL were pipetted on a microtiter plate, in duplicates. 50 μL of liquid *para*-nitrophenyl phosphate ligand (Sigma-Aldrich N7653) was quickly added to all wells and the microtiter plate was immediately put in BioTek Eon/SynergyH1/ Cytation3 microplate reader, which measured the absorbance at 405 nm every minute for 1h, or as long as the pattern of the color formation was clear. The *Gen5* v. 2.09 software automatically determined the maximal rate of yellow color formation (V_0_) as mAU/min within 4 time points in the beginning of the assay. The alkaline phosphatase activity of the protein measured as V_0_ corresponds to the protein concentration of the AP-CBM1 fusion protein. The loss of alkaline phosphatase activity between the control and sample was interpreted to represent the part of the protein bound to the ligand.

## Supporting information

S1 Appendix Protein sequence data. (DOCX)

S1 Fig Cartoon representation of the crystal structure of the CBM1 domain of Cel7A from *T*. *reesei*. The disulfide bridges are shown in yellow, and the tyrosines involved in cellulose binding in green. Image created with Chimera, PDB ID: 1CBH.
(TIF)

S2 Fig **Homology models of the (A) EHUX2, (B) EHUX4 and (C) EHUX5 CBM1 domains, displayed as blue ribbons, with Cel7A-CBM1 as overlay, displayed in red**. Cysteine bridges and the side-chains of residues that are important for binding are shown. Homology models were created by SWISS-MODEL with the crystal structure of Cel7A-CBM1 (PDB ID: 1CBH) as a template.
(TIF)

S3 Fig Visual overview of the pelB-AP-CBM1-6His constructs cloned, expressed and characterized in this paper. The proteins were designed to contain an N-terminal pelB signal peptide, Alkaline Phosphatase (AP) as a carrier protein and the CBM1 of interest with a His-tag on the C-terminus. The spacers flanking the CBM1 domains can be cleaved by trypsin, which does not degrade the Cel7A-CBM1 (it does degrade most of the EHUX-CBM1s).
(TIF)

S1 Table Overview of the CBM1-containing proteins found in *E*. *huxleyi*, displaying their gene identifiers (GI) and predicted functions. The associated functions (of the proteins, besides their CBM1 domains) were obtained with Gene Ontology (GO) and Interpro, so they are predicted based on sequence, using protein domains and families. The proteins can be found in Uniprot database using their GI.
(DOCX)
