# Supplementary material for: Fungal-type carbohydrate binding modules from the coccolithophore Emiliania huxleyi show binding affinity to cellulose and chitin
Source: PLoS One. 2018 May 21;13(5):e0197875. doi: 10.1371/journal.pone.0197875 (PMC5962083; doi:10.1371/journal.pone.0197875)
Supplement: S1 Appendix — (DOCX) [file pone.0197875.s001.docx]

**Fungal-type Carbohydrate Binding Modules from the coccolithophore *Emiliania huxleyi* show binding affinity to cellulose and chitin**

**Bart J.M. Rooijakkers^1^, Martina S. Ikonen^1^, Markus B. Linder^1*^**

**Supporting Information**

^1^ Department of Bioproducts and Biosystems, School of Chemical Engineering, Aalto University, Espoo, Finland.

^*^ Corresponding author (email: [markus.linder@aalto.fi](mailto:markus.linder@aalto.fi)) (ML)

**Protein sequences data**

The sequences below are annotated as follows:

pelB signal peptide: underlined

*Alkaline Phosphatase*: italic

**6His purification tag**: bold

CBM1: green

**pelB-AP-C7A-CBM1 (532 AA – 55.4 kDa)**

MKYLLPTAAAGLLLLAAQPAMASR*TPEMPVLENRAAQGDITAPGGARRLTGDQTAALRDSLSDKPAKNIILLIGDGMGDSEITAARNYAEGAGGFFKGIDALPLTGQYTHYALNKKTGKPDYVTDSAASATAWSTGVKTYNGALGVDIHEKDHPTILEMAKAAGLATGNVSTAELQDATPAALVAHVTSRKCYGPSATSEKCPGNALEKGGKGSITEQLLNARADVTLGGGAKTFAETATAGEWQGKTLREQAQARGYQLVSDAASLNSVTEANQQKPLLGLFADGNMPVRWLGPKATYHGNIDKPAVTCTPNPQRNDSVPTLAQMTDKAIELLSKNEKGFFLQVEGASIDKQDHAANPCGQIGETVDLDEAVQRALEFAKKEGNTLVIVTADHAHASQIVAPDTKAPGLTQALNTKDGAVMVMSYGNSEEDSQEHTGSQLRIAAYGPHAANVVGLTDQTDLFYTMKAALGLK*GGGGSKGGGSPTQSHYGQCGGIGYSGPTVCASGTTCQVLNPYYSQCLPGGKGE**HHHHHH**

**pelB-AP-EHUX1a (540 AA – 56.7 kDa)**

MKYLLPTAAAGLLLLAAQPAMASR*TPEMPVLENRAAQGDITAPGGARRLTGDQTAALRDSLSDKPAKNIILLIGDGMGDSEITAARNYAEGAGGFFKGIDALPLTGQYTHYALNKKTGKPDYVTDSAASATAWSTGVKTYNGALGVDIHEKDHPTILEMAKAAGLATGNVSTAELQDATPAALVAHVTSRKCYGPSATSEKCPGNALEKGGKGSITEQLLNARADVTLGGGAKTFAETATAGEWQGKTLREQAQARGYQLVSDAASLNSVTEANQQKPLLGLFADGNMPVRWLGPKATYHGNIDKPAVTCTPNPQRNDSVPTLAQMTDKAIELLSKNEKGFFLQVEGASIDKQDHAANPCGQIGETVDLDEAVQRALEFAKKEGNTLVIVTADHAHASQIVAPDTKAPGLTQALNTKDGAVMVMSYGNSEEDSQEHTGSQLRIAAYGPHAANVVGLTDQTDLFYTMKAALGLK*GGGGSKGGGSVAEAEAWEQCGGSDWTGPKQCPMDHTCLVRREKFSQCVPVWLDDWGGSKGE**HHHHHH**

**pelB-AP-EHUX1b (540 AA – 56.8 kDa)**

MKYLLPTAAAGLLLLAAQPAMASR*TPEMPVLENRAAQGDITAPGGARRLTGDQTAALRDSLSDKPAKNIILLIGDGMGDSEITAARNYAEGAGGFFKGIDALPLTGQYTHYALNKKTGKPDYVTDSAASATAWSTGVKTYNGALGVDIHEKDHPTILEMAKAAGLATGNVSTAELQDATPAALVAHVTSRKCYGPSATSEKCPGNALEKGGKGSITEQLLNARADVTLGGGAKTFAETATAGEWQGKTLREQAQARGYQLVSDAASLNSVTEANQQKPLLGLFADGNMPVRWLGPKATYHGNIDKPAVTCTPNPQRNDSVPTLAQMTDKAIELLSKNEKGFFLQVEGASIDKQDHAANPCGQIGETVDLDEAVQRALEFAKKEGNTLVIVTADHAHASQIVAPDTKAPGLTQALNTKDGAVMVMSYGNSEEDSQEHTGSQLRIAAYGPHAANVVGLTDQTDLFYTMKAALGLK*GGGGSKGGGSPRNPGPWEQCGGKSYEGPTACPREYTCQYRRETFSQCIPQFIAKWGGSKGE**HHHHHH**

**pelB-AP-EHUX2 (539 AA – 56.3 kDa)**

MKYLLPTAAAGLLLLAAQPAMASR*TPEMPVLENRAAQGDITAPGGARRLTGDQTAALRDSLSDKPAKNIILLIGDGMGDSEITAARNYAEGAGGFFKGIDALPLTGQYTHYALNKKTGKPDYVTDSAASATAWSTGVKTYNGALGVDIHEKDHPTILEMAKAAGLATGNVSTAELQDATPAALVAHVTSRKCYGPSATSEKCPGNALEKGGKGSITEQLLNARADVTLGGGAKTFAETATAGEWQGKTLREQAQARGYQLVSDAASLNSVTEANQQKPLLGLFADGNMPVRWLGPKATYHGNIDKPAVTCTPNPQRNDSVPTLAQMTDKAIELLSKNEKGFFLQVEGASIDKQDHAANPCGQIGETVDLDEAVQRALEFAKKEGNTLVIVTADHAHASQIVAPDTKAPGLTQALNTKDGAVMVMSYGNSEEDSQEHTGSQLRIAAYGPHAANVVGLTDQTDLFYTMKAALGLK*GGGGSKGGGSTICQLKWAQCGGNSWTGATCCEAGSVCTADSAWYSQCKPSRNYLGGSKGE**HHHHHH**

**pelB-AP-EHUX3 (537 AA – 56.0 kDa)**

MKYLLPTAAAGLLLLAAQPAMASR*TPEMPVLENRAAQGDITAPGGARRLTGDQTAALRDSLSDKPAKNIILLIGDGMGDSEITAARNYAEGAGGFFKGIDALPLTGQYTHYALNKKTGKPDYVTDSAASATAWSTGVKTYNGALGVDIHEKDHPTILEMAKAAGLATGNVSTAELQDATPAALVAHVTSRKCYGPSATSEKCPGNALEKGGKGSITEQLLNARADVTLGGGAKTFAETATAGEWQGKTLREQAQARGYQLVSDAASLNSVTEANQQKPLLGLFADGNMPVRWLGPKATYHGNIDKPAVTCTPNPQRNDSVPTLAQMTDKAIELLSKNEKGFFLQVEGASIDKQDHAANPCGQIGETVDLDEAVQRALEFAKKEGNTLVIVTADHAHASQIVAPDTKAPGLTQALNTKDGAVMVMSYGNSEEDSQEHTGSQLRIAAYGPHAANVVGLTDQTDLFYTMKAALGLK*GGGGSKGGGSAGEALAWGQCGGRRPTDTRPCADGALCIVRNERTNFSQCVPSGGSKGE**HHHHHH**

**pelB-AP-EHUX4 (539 AA – 56.2 kDa)**

MKYLLPTAAAGLLLLAAQPAMASR*TPEMPVLENRAAQGDITAPGGARRLTGDQTAALRDSLSDKPAKNIILLIGDGMGDSEITAARNYAEGAGGFFKGIDALPLTGQYTHYALNKKTGKPDYVTDSAASATAWSTGVKTYNGALGVDIHEKDHPTILEMAKAAGLATGNVSTAELQDATPAALVAHVTSRKCYGPSATSEKCPGNALEKGGKGSITEQLLNARADVTLGGGAKTFAETATAGEWQGKTLREQAQARGYQLVSDAASLNSVTEANQQKPLLGLFADGNMPVRWLGPKATYHGNIDKPAVTCTPNPQRNDSVPTLAQMTDKAIELLSKNEKGFFLQVEGASIDKQDHAANPCGQIGETVDLDEAVQRALEFAKKEGNTLVIVTADHAHASQIVAPDTKAPGLTQALNTKDGAVMVMSYGNSEEDSQEHTGSQLRIAAYGPHAANVVGLTDQTDLFYTMKAALGLK*GGGGSKGGGSTICQLKWAQCGGNSWTGATCCEAGSVCTASSAWYSQCKPSRNYLGGSKGE**HHHHHH**

**pelB-AP-EHUX5 (538 AA – 56.3 kDa)**

MKYLLPTAAAGLLLLAAQPAMASR*TPEMPVLENRAAQGDITAPGGARRLTGDQTAALRDSLSDKPAKNIILLIGDGMGDSEITAARNYAEGAGGFFKGIDALPLTGQYTHYALNKKTGKPDYVTDSAASATAWSTGVKTYNGALGVDIHEKDHPTILEMAKAAGLATGNVSTAELQDATPAALVAHVTSRKCYGPSATSEKCPGNALEKGGKGSITEQLLNARADVTLGGGAKTFAETATAGEWQGKTLREQAQARGYQLVSDAASLNSVTEANQQKPLLGLFADGNMPVRWLGPKATYHGNIDKPAVTCTPNPQRNDSVPTLAQMTDKAIELLSKNEKGFFLQVEGASIDKQDHAANPCGQIGETVDLDEAVQRALEFAKKEGNTLVIVTADHAHASQIVAPDTKAPGLTQALNTKDGAVMVMSYGNSEEDSQEHTGSQLRIAAYGPHAANVVGLTDQTDLFYTMKAALGLK*GGGGSKGGGSDYPGLTWHAQCGGVGFSTDPLACQEGLGCYQRNEYYWQCLEAPGGSKGE**HHHHHH**

**Protein sequences of CBM1-containing *E. huxleyi* genes**

Every sequence is showin fasta format and the titles include a link to their Uniprot entry.

**EHUX1a & EHUX1b: R1C3S1 (573 AA)** [*http://www.uniprot.org/uniprot/R1C0H5*](http://www.uniprot.org/uniprot/R1C0H5)

>tr|R1C3S1|R1C3S1_EMIHU Uncharacterized protein OS=Emiliania huxleyi GN=EMIHUDRAFT_102755 PE=4 SV=1

MALAALAAVAVAVAVAVGAAAAAAAAAAAGGLAVPEMAVAAVAMVVAVGAAAAAAAGGLA

VPEMAVAAVAMVVAVGAAAAAATATAGGLAVLEMAVAAVAMVVAVGAAAAAATATAGGLA

VLEMAVAAVAMVVAVGAAAAAATATAGGLAVLEMAVAAVAMVVAVGAAAAAAAAAVAAAA

GGLAVPEMALALAAAVAMVVAVEVAVGAAVVEVMEEVEVEVVEVVEVVAVKSLAVARAVA

AVAVAVAEAEAWEQCGGSDWTGPKQCPMDHTCLVRREKFSQCVPVWLDDWLGWRTGDGAI

YDDGPADDIVAHLAKPMHDSKSPPRNPGPWEQCGGKSYEGPTACPREYTCQYRRETFSQC

IPQFIAKWIPSHPAHDNDDADQKHRLGSDDASSDDDDADRPDTDDKHDPEKPSDDDDVDS

KDDDDKHDPDETRDDDKHADEDGEKDSAHSHDDADKALACRRRQPFPACVCAASDSALAS

PQHRHGSDDDDADRPDKDDKHDPEKPSDDDDVDSKDDDDKHDPDETRDDDKHADSKHHND

YGSSDADDDTDSKHDDDADKDGKHANDSDDDSK

**EHUX2: R1C6A1 (602 AA***)* [*http://www.uniprot.org/uniprot/R1C6A1*](http://www.uniprot.org/uniprot/R1C6A1)

>tr|R1C6A1|R1C6A1_EMIHU Uncharacterized protein OS=Emiliania huxleyi GN=EMIHUDRAFT_464128 PE=4 SV=1

MSRTQSSEYLPMPSFRLMADYSSPPLEDDVLGEAVPLSALKPLAQLGAGEFCTVHRATLR

GSMVALKRLRPARLALGDSSRLDLGREIAVLSRVEHPNIIRLVAHGVDGDGVPFCCLEMF

SQPLSALLPSGAEVGFFARQREARKWPLARGLSVGLQLAEALRYCHDEFMPGYRLLHRDL

KPANVGIMPDGRAVLADFGVLSLWKRDAADDGDVRKLTGVTGSLRYMAPEAEVFSFTSLL

YHLLSRRKPFALMTPEMFLREACRGGKRPPLAKAWPLPLRSLMAASWSAKHSVRFWARRV

MLVPLVVTVLTQPAPLAEKATEAAVSAQPIPPTEKAAGEAAPPTEKWVTGGCDWRGCAHT

NHLTGTTTLTDYKGGKITTNKWTGQTTASNHQGSYTANKYTGQYKAEGVNGKIEGNHHTG

DWQAQGCNGWGCGTSGGNHHSGDWWATPHPAPGGNPTVCCMAMTASCMACQKGVSLSAWC

AAHPGYAGCPTAVFPRPGMPGGNPTICQLKWAQCGGNSWTGATCCEAGSVCTADSAWYSQ

CKPSRNYLRKIDEAGELVATHAGVFAATGAFAAIFLAFGLLRRSRASKPDAATAGAPAMQ

LV

**EHUX3: R1EZE3 (400 A)** [*http://www.uniprot.org/uniprot/R1EZE3*](http://www.uniprot.org/uniprot/R1EZE3)

>tr|R1EZE3|R1EZE3_EMIHU Uncharacterized protein OS=Emiliania huxleyi GN=EMIHUDRAFT_236834 PE=4 SV=1

MHDKRRTNEARSLSDMRRVALASAALAFCLPAQWVADASAAVACLTAATGRRLGASARLG

VAIVASATRLDSTLSQELPHALARVMAHATRLNARLASRKCDPSAPSNDRPSPSATRPAP

LIMLGPASDGPAVSVGLLVEAPERSRQRRLRWLGAAAALALHAGLLLVFYASGGSLSAPS

LRQRFDAYDGDGDGRITTDDARQLLLDSDVCCPTPEAAAEAVAEMDSDVDGRTYARNVAV

GHVEWGEFKLSLDRPVTVAKPATRAADSGGADERTDVPSAGEALAWGQCGGRRPTDTRPC

ADGALCIVRNERTNFSQCVPSNPQRDPSMNAPGDSAGGQAGSDEPPRRATPSGPLDIGGA

PATYDDPDFAALGTTHGDPARFPRWLLLVMTAVVMPGDTA

**EHUX4: R1EB44 (266 AA)** [*http://www.uniprot.org/uniprot/R1EB44*](http://www.uniprot.org/uniprot/R1EB44)

>tr|R1EB44|R1EB44_EMIHU Uncharacterized protein OS=Emiliania huxleyi GN=EMIHUDRAFT_447941 PE=4 SV=1

MLVPLVLTVLTQPAPLATEAVSVQPTPPTERAAGEAAPPTEKAAGEAAPPTEKWISSSCS

LSGCVHTNYLTGTTTAHDFKGGKVTTNHWTGETTASNHQGSITSNKYTGDWKAHGCNSWG

CGTSGGNHRTGDYWSKGVSLSAWCAAHPGYAGCPTAVFPRPGMPGGNPTICQLKWAQCGG

NSWTGATCCEAGSVCTASSAWYSQCKPSRNYLHKIDEAGELVATHAGVFAATGAFAAVFL

AVGLLRRSRASRPEAATAEAPAVQLV

**EHUX5: R1CQK6 (878 AA)** [*http://www.uniprot.org/uniprot/R1CQK6*](http://www.uniprot.org/uniprot/R1CQK6)

>tr|R1CQK6|R1CQK6_EMIHU Uncharacterized protein OS=Emiliania huxleyi GN=EMIHUDRAFT_237928 PE=4 SV=1

MSPGWCNMASHCYATCKPWMTRCSAPEPTVPPCSSNAVETWGKCSYSGQCCSSADECFIK

TVFKPYAKNSYYAQCRPSCPANNDPIWACASGRVAPSREEFEKDYPGLTWHAQCGGVGFS

TDPLACQEGLGCYQRNEYYWQCLEAPAGACQSFDEASERATLSDIIKVPESNIRVALRGC

STSGRRQMQSSDSDTTPEAVISAAQNSDHIALNEPGGSCTDHCASLGTTCADETPNNPPL

PADEWPKTVWPTTEAQMEAISDAWGLGCTAYVDRVLILDGNSDTEACVHDAEATTHGGTA

IAAQCCDGSTCKRRTSGSNDDCIAGLWSSSFQYTTFQEASARCAALGYTLCDKNCQGTGC

SYDSIWACVHDAEATTHGGTAIAAQCCDGSTCKRRTSGSNDDCIAGLWSSSFQYTTFQEA

SARCAALGYTLCDKNCQGTGCSYDSIWGGSCTDHCASLGTTCADETPNNPPLPADEWPKT

VWPTTEAQMEAISDAWGLGCTTYVDRVLILDGNSDTEACVHDAEATTHGGTAIAAQCCDG

STCKRRTSGSNDDCIAGLWSSSFQYTTFQEASARCAALGYTLCDKNCQGTGCSYDSIWAC

VHDAEATTHGGTAIAAQCCDGSTCKRRTSGSNDDCIAGLWSSSFQYTTFQEASARCAALG

YTLCDKNCQGTGCSYDSIWGGSCTDHCASLGTTCADETPNNPPLPADEWPKTVWPTTEAQ

MEAISDAWGLGCTTYVDRPDLYVRVDPPEGSTCEWCGKQDSGFGSSLGLDGSGGRAGQVW

TSPGGITYTSNPWLSCGLTSGFIYGPEGFPVAATLTYAIEKCSQLSISSGRECIGLRYTM

DLNSVRQQYLGCYGLYTDSQSMRGADDDPEYFIIPGSR

**Plasmid sequences**

**Empty expression plasmid pBR1a (5263 bp, pET28a(+) derivative for Golden gate cloning)**

TGGCGAATGGGACGCGCCCTGTAGCGGCGCATTAAGCGCGGCGGGTGTGGTGGTTACGCGCAGCGTGACCGCTACACTTGCCAGCGCCCTAGCGCCCGCTCCTTTCGCTTTCTTCCCTTCCTTTCTCGCCACGTTCGCCGGCTTTCCCCGTCAAGCTCTAAATCGGGGGCTCCCTTTAGGGTTCCGATTTAGTGCTTTACGGCACCTCGACCCCAAAAAACTTGATTAGGGTGATGGTTCACGTAGTGGGCCATCGCCCTGATAGACGGTTTTTCGCCCTTTGACGTTGGAGTCCACGTTCTTTAATAGTGGACTCTTGTTCCAAACTGGAACAACACTCAACCCTATCTCGGTCTATTCTTTTGATTTATAAGGGATTTTGCCGATTTCGGCCTATTGGTTAAAAAATGAGCTGATTTAACAAAAATTTAACGCGAATTTTAACAAAATATTAACGCTTACAATTTAGGTGGCACTTTTCGGGGAAATGTGCGCGGAACCCCTATTTGTTTATTTTTCTAAATACATTCAAATATGTATCCGCTCATGAATTAATTCTTAGAAAAACTCATCGAGCATCAAATGAAACTGCAATTTATTCATATCAGGATTATCAATACCATATTTTTGAAAAAGCCGTTTCTGTAATGAAGGAGAAAACTCACCGAGGCAGTTCCATAGGATGGCAAGATCCTGGTATCGGTCTGCGATTCCGACTCGTCCAACATCAATACAACCTATTAATTTCCCCTCGTCAAAAATAAGGTTATCAAGTGAGAAATCACCATGAGTGACGACTGAATCCGGTGAGAATGGCAAAAGTTTATGCATTTCTTTCCAGACTTGTTCAACAGGCCAGCCATTACGCTCGTCATCAAAATCACTCGCATCAACCAAACCGTTATTCATTCGTGATTGCGCCTGAGCGAGACGAAATACGCGATCGCTGTTAAAAGGACAATTACAAACAGGAATCGAATGCAACCGGCGCAGGAACACTGCCAGCGCATCAACAATATTTTCACCTGAATCAGGATATTCTTCTAATACCTGGAATGCTGTTTTCCCGGGGATCGCAGTGGTGAGTAACCATGCATCATCAGGAGTACGGATAAAATGCTTGATGGTCGGAAGAGGCATAAATTCCGTCAGCCAGTTTAGTCTGACCATCTCATCTGTAACATCATTGGCAACGCTACCTTTGCCATGTTTCAGAAACAACTCTGGCGCATCGGGCTTCCCATACAATCGATAGATTGTCGCACCTGATTGCCCGACATTATCGCGAGCCCATTTATACCCATATAAATCAGCATCCATGTTGGAATTTAATCGCGGCCTAGAGCAAGACGTTTCCCGTTGAATATGGCTCATAACACCCCTTGTATTACTGTTTATGTAAGCAGACAGTTTTATTGTTCATGACCAAAATCCCTTAACGTGAGTTTTCGTTCCACTGAGCGTCAGACCCCGTAGAAAAGATCAAAGGATCTTCTTGAGATCCTTTTTTTCTGCGCGTAATCTGCTGCTTGCAAACAAAAAAACCACCGCTACCAGCGGTGGTTTGTTTGCCGGATCAAGAGCTACCAACTCTTTTTCCGAAGGTAACTGGCTTCAGCAGAGCGCAGATACCAAATACTGTCCTTCTAGTGTAGCCGTAGTTAGGCCACCACTTCAAGAACTCTGTAGCACCGCCTACATACCTCGCTCTGCTAATCCTGTTACCAGTGGCTGCTGCCAGTGGCGATAAGTCGTGTCTTACCGGGTTGGACTCAAGACGATAGTTACCGGATAAGGCGCAGCGGTCGGGCTGAACGGGGGGTTCGTGCACACAGCCCAGCTTGGAGCGAACGACCTACACCGAACTGAGATACCTACAGCGTGAGCTATGAGAAAGCGCCACGCTTCCCGAAGGGAGAAAGGCGGACAGGTATCCGGTAAGCGGCAGGGTCGGAACAGGAGAGCGCACGAGGGAGCTTCCAGGGGGAAACGCCTGGTATCTTTATAGTCCTGTCGGGTTTCGCCACCTCTGACTTGAGCGTCGATTTTTGTGATGCTCGTCAGGGGGGCGGAGCCTATGGAAAAACGCCAGCAACGCGGCCTTTTTACGGTTCCTGGCCTTTTGCTGGCCTTTTGCTCACATGTTCTTTCCTGCGTTATCCCCTGATTCTGTGGATAACCGTATTACCGCCTTTGAGTGAGCTGATACCGCTCGCCGCAGCCGAACGACCGAGCGCAGCGAGTCAGTGAGCGAGGAAGCGGAAGAGCGCCTGATGCGGTATTTTCTCCTTACGCATCTGTGCGGTATTTCACACCGCATATATGGTGCACTCTCAGTACAATCTGCTCTGATGCCGCATAGTTAAGCCAGTATACACTCCGCTATCGCTACGTGACTGGGTCATGGCTGCGCCCCGACACCCGCCAACACCCGCTGACGCGCCCTGACGGGCTTGTCTGCTCCCGGCATCCGCTTACAGACAAGCTGTGACCGTCTCCGGGAGCTGCATGTGTCAGAGGTTTTCACCGTCATCACCGAAACGCGCGAGGCAGCTGCGGTAAAGCTCATCAGCGTGGTCGTGAAGCGATTCACAGATGTCTGCCTGTTCATCCGCGTCCAGCTCGTTGAGTTTCTCCAGAAGCGTTAATGTCTGGCTTCTGATAAAGCGGGCCATGTTAAGGGCGGTTTTTTCCTGTTTGGTCACTGATGCCTCCGTGTAAGGGGGATTTCTGTTCATGGGGGTAATGATACCGATGAAACGAGAGAGGATGCTCACGATACGGGTTACTGATGATGAACATGCCCGGTTACTGGAACGTTGTGAGGGTAAACAACTGGCGGTATGGATGCGGCGGGACCAGAGAAAAATCACTCAGGGTCAATGCCAGCGCTTCGTTAATACAGATGTAGGTGTTCCACAGGGTAGCCAGCAGCATCCTGCGATGCAGATCCGGAACATAATGGTGCAGGGCGCTGACTTCCGCGTTTCCAGACTTTACGAAACACGGAAACCGAAGACCATTCATGTTGTTGCTCAGGTCGCAGACGTTTTGCAGCAGCAGTCGCTTCACGTTCGCTCGCGTATCGGTGATTCATTCTGCTAACCAGTAAGGCAACCCCGCCAGCCTAGCCGGGTCCTCAACGACAGGAGCACGATCATGCGCACCCGTGGGGCCGCCATGCCGGCGATAATGGCCTGCTTCTCGCCGAAACGTTTGGTGGCGGGACCAGTGACGAAGGCTTGAGCGAGGGCGTGCAAGATTCCGAATACCGCAAGCGACAGGCCGATCATCGTCGCGCTCCAGCGAAAGCGGTCCTCGCCGAAAATGACCCAGAGCGCTGCCGGCACCTGTCCTACGAGTTGCATGATAAAGAAGACAGTCATAAGTGCGGCGACGATAGTCATGCCCCGCGCCCACCGGAAGGAGCTGACTGGGTTGAAGGCTCTCAAGGGCATCGGTCGAGATCCCGGTGCCTAATGAGTGAGCTAACTTACATTAATTGCGTTGCGCTCACTGCCCGCTTTCCAGTCGGGAAACCTGTCGTGCCAGCTGCATTAATGAATCGGCCAACGCGCGGGGAGAGGCGGTTTGCGTATTGGGCGCCAGGGTGGTTTTTCTTTTCACCAGTGAGACGGGCAACAGCTGATTGCCCTTCACCGCCTGGCCCTGAGAGAGTTGCAGCAAGCGGTCCACGCTGGTTTGCCCCAGCAGGCGAAAATCCTGTTTGATGGTGGTTAACGGCGGGATATAACATGAGCTGTCTTCGGTATCGTCGTATCCCACTACCGAGATATCCGCACCAACGCGCAGCCCGGACTCGGTAATGGCGCGCATTGCGCCCAGCGCCATCTGATCGTTGGCAACCAGCATCGCAGTGGGAACGATGCCCTCATTCAGCATTTGCATGGTTTGTTGAAAACCGGACATGGCACTCCAGTCGCCTTCCCGTTCCGCTATCGGCTGAATTTGATTGCGAGTGAGATATTTATGCCAGCCAGCCAGACGCAGACGCGCCGAGACAGAACTTAATGGGCCCGCTAACAGCGCGATTTGCTGGTGACCCAATGCGACCAGATGCTCCACGCCCAGTCGCGTACCGTCTTCATGGGAGAAAATAATACTGTTGATGGGTGTCTGGTCAGAGACATCAAGAAATAACGCCGGAACATTAGTGCAGGCAGCTTCCACAGCAATGGCATCCTGGTCATCCAGCGGATAGTTAATGATCAGCCCACTGACGCGTTGCGCGAGAAGATTGTGCACCGCCGCTTTACAGGCTTCGACGCCGCTTCGTTCTACCATCGACACCACCACGCTGGCACCCAGTTGATCGGCGCGAGATTTAATCGCCGCGACAATTTGCGACGGCGCGTGCAGGGCCAGACTGGAGGTGGCAACGCCAATCAGCAACGACTGTTTGCCCGCCAGTTGTTGTGCCACGCGGTTGGGAATGTAATTCAGCTCCGCCATCGCCGCTTCCACTTTTTCCCGCGTTTTCGCAGAAACGTGGCTGGCCTGGTTCACCACGCGGGAAACGGTCTGATAAGAGACACCGGCATACTCTGCGACATCGTATAACGTTACTGGTTTCACATTCACCACCCTGAATTGACTCTCTTCCGGGCGCTATCATGCCATACCGCGAAAGGTTTTGCGCCATTCGATGGTGTCCGGGATCTCGACGCTCTCCCTTATGCGACTCCTGCATTAGGAAGCAGCCCAGTAGTAGGTTGAGGCCGTTGAGCACCGCCGCCGCAAGGAATGGTGCATGCAAGGAGATGGCGCCCAACAGTCCCCCGGCCACGGGGCCTGCCACCATACCCACGCCGAAACAAGCGCTCATGAGCCCGAAGTGGCGAGCCCGATCTTCCCCATCGGTGATGTCGGCGATATAGGCGCCAGCAACCGCACCTGTGGCGCCGGTGATGCCGGCCACGATGCGTCCGGCGTAGAGGATCGAGATCTCGATCCCGCGAAATTAATACGACTCACTATAGGGGAATTGTGAGCGGATAACAATTCCCCTCTAGAAATAATTTTGTTTAACTTTAAGAAGGAGATATACCATGAGGAGACCGCGGATCCGAATTCGGGTCTCT*CGAG*CACCACCACCACCACCACTGAGATCCGGCTGCTAACAAAGCCCGAAAGGAAGCTGAGTTGGCTGCTGCCACCGCTGAGCAATAACTAGCATAACCCCTTGGGGCCTCTAAACGGGTCTTGAGGGGTTTTTTGCTGAAAGGAGGAACTATATCCGGAT

**pBR1b pelB-AP-Cel7a-CBM1 (6 804 bp)**

TGGCGAATGGGACGCGCCCTGTAGCGGCGCATTAAGCGCGGCGGGTGTGGTGGTTACGCGCAGCGTGACCGCTACACTTGCCAGCGCCCTAGCGCCCGCTCCTTTCGCTTTCTTCCCTTCCTTTCTCGCCACGTTCGCCGGCTTTCCCCGTCAAGCTCTAAATCGGGGGCTCCCTTTAGGGTTCCGATTTAGTGCTTTACGGCACCTCGACCCCAAAAAACTTGATTAGGGTGATGGTTCACGTAGTGGGCCATCGCCCTGATAGACGGTTTTTCGCCCTTTGACGTTGGAGTCCACGTTCTTTAATAGTGGACTCTTGTTCCAAACTGGAACAACACTCAACCCTATCTCGGTCTATTCTTTTGATTTATAAGGGATTTTGCCGATTTCGGCCTATTGGTTAAAAAATGAGCTGATTTAACAAAAATTTAACGCGAATTTTAACAAAATATTAACGCTTACAATTTAGGTGGCACTTTTCGGGGAAATGTGCGCGGAACCCCTATTTGTTTATTTTTCTAAATACATTCAAATATGTATCCGCTCATGAATTAATTCTTAGAAAAACTCATCGAGCATCAAATGAAACTGCAATTTATTCATATCAGGATTATCAATACCATATTTTTGAAAAAGCCGTTTCTGTAATGAAGGAGAAAACTCACCGAGGCAGTTCCATAGGATGGCAAGATCCTGGTATCGGTCTGCGATTCCGACTCGTCCAACATCAATACAACCTATTAATTTCCCCTCGTCAAAAATAAGGTTATCAAGTGAGAAATCACCATGAGTGACGACTGAATCCGGTGAGAATGGCAAAAGTTTATGCATTTCTTTCCAGACTTGTTCAACAGGCCAGCCATTACGCTCGTCATCAAAATCACTCGCATCAACCAAACCGTTATTCATTCGTGATTGCGCCTGAGCGAGACGAAATACGCGATCGCTGTTAAAAGGACAATTACAAACAGGAATCGAATGCAACCGGCGCAGGAACACTGCCAGCGCATCAACAATATTTTCACCTGAATCAGGATATTCTTCTAATACCTGGAATGCTGTTTTCCCGGGGATCGCAGTGGTGAGTAACCATGCATCATCAGGAGTACGGATAAAATGCTTGATGGTCGGAAGAGGCATAAATTCCGTCAGCCAGTTTAGTCTGACCATCTCATCTGTAACATCATTGGCAACGCTACCTTTGCCATGTTTCAGAAACAACTCTGGCGCATCGGGCTTCCCATACAATCGATAGATTGTCGCACCTGATTGCCCGACATTATCGCGAGCCCATTTATACCCATATAAATCAGCATCCATGTTGGAATTTAATCGCGGCCTAGAGCAAGACGTTTCCCGTTGAATATGGCTCATAACACCCCTTGTATTACTGTTTATGTAAGCAGACAGTTTTATTGTTCATGACCAAAATCCCTTAACGTGAGTTTTCGTTCCACTGAGCGTCAGACCCCGTAGAAAAGATCAAAGGATCTTCTTGAGATCCTTTTTTTCTGCGCGTAATCTGCTGCTTGCAAACAAAAAAACCACCGCTACCAGCGGTGGTTTGTTTGCCGGATCAAGAGCTACCAACTCTTTTTCCGAAGGTAACTGGCTTCAGCAGAGCGCAGATACCAAATACTGTCCTTCTAGTGTAGCCGTAGTTAGGCCACCACTTCAAGAACTCTGTAGCACCGCCTACATACCTCGCTCTGCTAATCCTGTTACCAGTGGCTGCTGCCAGTGGCGATAAGTCGTGTCTTACCGGGTTGGACTCAAGACGATAGTTACCGGATAAGGCGCAGCGGTCGGGCTGAACGGGGGGTTCGTGCACACAGCCCAGCTTGGAGCGAACGACCTACACCGAACTGAGATACCTACAGCGTGAGCTATGAGAAAGCGCCACGCTTCCCGAAGGGAGAAAGGCGGACAGGTATCCGGTAAGCGGCAGGGTCGGAACAGGAGAGCGCACGAGGGAGCTTCCAGGGGGAAACGCCTGGTATCTTTATAGTCCTGTCGGGTTTCGCCACCTCTGACTTGAGCGTCGATTTTTGTGATGCTCGTCAGGGGGGCGGAGCCTATGGAAAAACGCCAGCAACGCGGCCTTTTTACGGTTCCTGGCCTTTTGCTGGCCTTTTGCTCACATGTTCTTTCCTGCGTTATCCCCTGATTCTGTGGATAACCGTATTACCGCCTTTGAGTGAGCTGATACCGCTCGCCGCAGCCGAACGACCGAGCGCAGCGAGTCAGTGAGCGAGGAAGCGGAAGAGCGCCTGATGCGGTATTTTCTCCTTACGCATCTGTGCGGTATTTCACACCGCATATATGGTGCACTCTCAGTACAATCTGCTCTGATGCCGCATAGTTAAGCCAGTATACACTCCGCTATCGCTACGTGACTGGGTCATGGCTGCGCCCCGACACCCGCCAACACCCGCTGACGCGCCCTGACGGGCTTGTCTGCTCCCGGCATCCGCTTACAGACAAGCTGTGACCGTCTCCGGGAGCTGCATGTGTCAGAGGTTTTCACCGTCATCACCGAAACGCGCGAGGCAGCTGCGGTAAAGCTCATCAGCGTGGTCGTGAAGCGATTCACAGATGTCTGCCTGTTCATCCGCGTCCAGCTCGTTGAGTTTCTCCAGAAGCGTTAATGTCTGGCTTCTGATAAAGCGGGCCATGTTAAGGGCGGTTTTTTCCTGTTTGGTCACTGATGCCTCCGTGTAAGGGGGATTTCTGTTCATGGGGGTAATGATACCGATGAAACGAGAGAGGATGCTCACGATACGGGTTACTGATGATGAACATGCCCGGTTACTGGAACGTTGTGAGGGTAAACAACTGGCGGTATGGATGCGGCGGGACCAGAGAAAAATCACTCAGGGTCAATGCCAGCGCTTCGTTAATACAGATGTAGGTGTTCCACAGGGTAGCCAGCAGCATCCTGCGATGCAGATCCGGAACATAATGGTGCAGGGCGCTGACTTCCGCGTTTCCAGACTTTACGAAACACGGAAACCGAAGACCATTCATGTTGTTGCTCAGGTCGCAGACGTTTTGCAGCAGCAGTCGCTTCACGTTCGCTCGCGTATCGGTGATTCATTCTGCTAACCAGTAAGGCAACCCCGCCAGCCTAGCCGGGTCCTCAACGACAGGAGCACGATCATGCGCACCCGTGGGGCCGCCATGCCGGCGATAATGGCCTGCTTCTCGCCGAAACGTTTGGTGGCGGGACCAGTGACGAAGGCTTGAGCGAGGGCGTGCAAGATTCCGAATACCGCAAGCGACAGGCCGATCATCGTCGCGCTCCAGCGAAAGCGGTCCTCGCCGAAAATGACCCAGAGCGCTGCCGGCACCTGTCCTACGAGTTGCATGATAAAGAAGACAGTCATAAGTGCGGCGACGATAGTCATGCCCCGCGCCCACCGGAAGGAGCTGACTGGGTTGAAGGCTCTCAAGGGCATCGGTCGAGATCCCGGTGCCTAATGAGTGAGCTAACTTACATTAATTGCGTTGCGCTCACTGCCCGCTTTCCAGTCGGGAAACCTGTCGTGCCAGCTGCATTAATGAATCGGCCAACGCGCGGGGAGAGGCGGTTTGCGTATTGGGCGCCAGGGTGGTTTTTCTTTTCACCAGTGAGACGGGCAACAGCTGATTGCCCTTCACCGCCTGGCCCTGAGAGAGTTGCAGCAAGCGGTCCACGCTGGTTTGCCCCAGCAGGCGAAAATCCTGTTTGATGGTGGTTAACGGCGGGATATAACATGAGCTGTCTTCGGTATCGTCGTATCCCACTACCGAGATATCCGCACCAACGCGCAGCCCGGACTCGGTAATGGCGCGCATTGCGCCCAGCGCCATCTGATCGTTGGCAACCAGCATCGCAGTGGGAACGATGCCCTCATTCAGCATTTGCATGGTTTGTTGAAAACCGGACATGGCACTCCAGTCGCCTTCCCGTTCCGCTATCGGCTGAATTTGATTGCGAGTGAGATATTTATGCCAGCCAGCCAGACGCAGACGCGCCGAGACAGAACTTAATGGGCCCGCTAACAGCGCGATTTGCTGGTGACCCAATGCGACCAGATGCTCCACGCCCAGTCGCGTACCGTCTTCATGGGAGAAAATAATACTGTTGATGGGTGTCTGGTCAGAGACATCAAGAAATAACGCCGGAACATTAGTGCAGGCAGCTTCCACAGCAATGGCATCCTGGTCATCCAGCGGATAGTTAATGATCAGCCCACTGACGCGTTGCGCGAGAAGATTGTGCACCGCCGCTTTACAGGCTTCGACGCCGCTTCGTTCTACCATCGACACCACCACGCTGGCACCCAGTTGATCGGCGCGAGATTTAATCGCCGCGACAATTTGCGACGGCGCGTGCAGGGCCAGACTGGAGGTGGCAACGCCAATCAGCAACGACTGTTTGCCCGCCAGTTGTTGTGCCACGCGGTTGGGAATGTAATTCAGCTCCGCCATCGCCGCTTCCACTTTTTCCCGCGTTTTCGCAGAAACGTGGCTGGCCTGGTTCACCACGCGGGAAACGGTCTGATAAGAGACACCGGCATACTCTGCGACATCGTATAACGTTACTGGTTTCACATTCACCACCCTGAATTGACTCTCTTCCGGGCGCTATCATGCCATACCGCGAAAGGTTTTGCGCCATTCGATGGTGTCCGGGATCTCGACGCTCTCCCTTATGCGACTCCTGCATTAGGAAGCAGCCCAGTAGTAGGTTGAGGCCGTTGAGCACCGCCGCCGCAAGGAATGGTGCATGCAAGGAGATGGCGCCCAACAGTCCCCCGGCCACGGGGCCTGCCACCATACCCACGCCGAAACAAGCGCTCATGAGCCCGAAGTGGCGAGCCCGATCTTCCCCATCGGTGATGTCGGCGATATAGGCGCCAGCAACCGCACCTGTGGCGCCGGTGATGCCGGCCACGATGCGTCCGGCGTAGAGGATCGAGATCTCGATCCCGCGAAATTAATACGACTCACTATAGGGGAATTGTGAGCGGATAACAATTCCCCTCTAGAAATAATTTTGTTTAACTTTAAGAAGGAGATATACCATGAAATACCTATTGCCTACGGCAGCCGCTGGATTGTTATTACTCGCGGCCCAGCCGGCCATGGCATCTAGGACTCCGGAAATGCCGGTTCTGGAAAATCGTGCAGCACAGGGTGATATTACCGCACCGGGTGGTGCACGTCGTCTGACCGGTGATCAGACCGCAGCACTGCGTGATAGCCTGAGCGATAAACCGGCAAAAAACATTATTCTGCTGATTGGTGATGGCATGGGTGATAGCGAAATTACCGCAGCACGTAATTATGCCGAAGGTGCCGGTGGTTTTTTTAAAGGTATTGATGCACTGCCGCTGACAGGTCAGTATACCCATTATGCACTGAATAAAAAAACCGGCAAACCGGATTATGTTACCGATAGCGCAGCAAGCGCAACCGCATGGTCAACCGGTGTTAAAACCTATAATGGTGCACTGGGTGTGGATATTCATGAAAAAGATCATCCGACCATTCTGGAAATGGCAAAAGCAGCAGGTCTGGCAACCGGTAATGTTAGCACCGCAGAACTGCAGGATGCAACACCGGCAGCACTGGTTGCACATGTTACCAGCCGTAAATGTTATGGTCCGAGCGCAACCAGCGAAAAATGTCCGGGTAATGCACTGGAAAAAGGTGGTAAAGGTAGCATTACCGAACAGCTGCTGAATGCACGTGCAGATGTTACCCTGGGTGGTGGTGCAAAAACCTTTGCAGAAACCGCAACCGCAGGCGAATGGCAGGGTAAAACCCTGCGTGAACAGGCACAGGCACGTGGTTATCAGCTGGTTAGTGATGCAGCAAGCCTGAATAGCGTTACCGAAGCAAATCAGCAGAAACCGCTGCTGGGTCTGTTTGCAGATGGTAATATGCCGGTTCGTTGGCTGGGTCCGAAAGCAACCTATCATGGTAATATTGATAAACCGGCTGTTACCTGTACCCCGAATCCGCAGCGTAATGATAGCGTTCCGACCCTGGCACAGATGACCGATAAAGCAATTGAACTGCTGAGCAAAAATGAAAAAGGCTTTTTTCTGCAGGTTGAAGGTGCCAGCATTGATAAACAGGATCATGCAGCAAATCCGTGTGGTCAGATTGGTGAAACCGTTGATCTGGATGAAGCAGTTCAGCGTGCACTGGAATTTGCAAAAAAAGAAGGTAATACCCTGGTTATTGTGACCGCAGATCATGCACATGCAAGCCAGATTGTTGCACCGGATACCAAAGCACCGGGTCTGACCCAGGCACTGAATACCAAAGATGGTGCAGTTATGGTTATGAGCTATGGTAATAGCGAAGAAGATAGCCAGGAACATACCGGTAGCCAGCTGCGTATTGCAGCATATGGTCCGCATGCAGCCAATGTTGTTGGTCTGACCGATCAAACCGACCTGTTTTATACCATGAAAGCAGCACTGGGTCTGAAAGGCGGCGGTGGTAGCAAAGGTGGTGGCTCTCCGACCCAGAGCCATTATGGTCAGTGTGGTGGTATTGGTTATAGCGGTCCGACCGTTTGTGCAAGCGGCACCACCTGTCAGGTTCTGAATCCGTATTATTCACAGTGTCTGCCTGGTGGTAAAGGCGAGCACCACCACCACCACCACTGAGATCCGGCTGCTAACAAAGCCCGAAAGGAAGCTGAGTTGGCTGCTGCCACCGCTGAGCAATAACTAGCATAACCCCTTGGGGCCTCTAAACGGGTCTTGAGGGGTTTTTTGCTGAAAGGAGGAACTATATCCGGAT

**pBR3g pelB-AP-EHUX1a**

TGGCGAATGGGACGCGCCCTGTAGCGGCGCATTAAGCGCGGCGGGTGTGGTGGTTACGCGCAGCGTGACCGCTACACTTGCCAGCGCCCTAGCGCCCGCTCCTTTCGCTTTCTTCCCTTCCTTTCTCGCCACGTTCGCCGGCTTTCCCCGTCAAGCTCTAAATCGGGGGCTCCCTTTAGGGTTCCGATTTAGTGCTTTACGGCACCTCGACCCCAAAAAACTTGATTAGGGTGATGGTTCACGTAGTGGGCCATCGCCCTGATAGACGGTTTTTCGCCCTTTGACGTTGGAGTCCACGTTCTTTAATAGTGGACTCTTGTTCCAAACTGGAACAACACTCAACCCTATCTCGGTCTATTCTTTTGATTTATAAGGGATTTTGCCGATTTCGGCCTATTGGTTAAAAAATGAGCTGATTTAACAAAAATTTAACGCGAATTTTAACAAAATATTAACGCTTACAATTTAGGTGGCACTTTTCGGGGAAATGTGCGCGGAACCCCTATTTGTTTATTTTTCTAAATACATTCAAATATGTATCCGCTCATGAATTAATTCTTAGAAAAACTCATCGAGCATCAAATGAAACTGCAATTTATTCATATCAGGATTATCAATACCATATTTTTGAAAAAGCCGTTTCTGTAATGAAGGAGAAAACTCACCGAGGCAGTTCCATAGGATGGCAAGATCCTGGTATCGGTCTGCGATTCCGACTCGTCCAACATCAATACAACCTATTAATTTCCCCTCGTCAAAAATAAGGTTATCAAGTGAGAAATCACCATGAGTGACGACTGAATCCGGTGAGAATGGCAAAAGTTTATGCATTTCTTTCCAGACTTGTTCAACAGGCCAGCCATTACGCTCGTCATCAAAATCACTCGCATCAACCAAACCGTTATTCATTCGTGATTGCGCCTGAGCGAGACGAAATACGCGATCGCTGTTAAAAGGACAATTACAAACAGGAATCGAATGCAACCGGCGCAGGAACACTGCCAGCGCATCAACAATATTTTCACCTGAATCAGGATATTCTTCTAATACCTGGAATGCTGTTTTCCCGGGGATCGCAGTGGTGAGTAACCATGCATCATCAGGAGTACGGATAAAATGCTTGATGGTCGGAAGAGGCATAAATTCCGTCAGCCAGTTTAGTCTGACCATCTCATCTGTAACATCATTGGCAACGCTACCTTTGCCATGTTTCAGAAACAACTCTGGCGCATCGGGCTTCCCATACAATCGATAGATTGTCGCACCTGATTGCCCGACATTATCGCGAGCCCATTTATACCCATATAAATCAGCATCCATGTTGGAATTTAATCGCGGCCTAGAGCAAGACGTTTCCCGTTGAATATGGCTCATAACACCCCTTGTATTACTGTTTATGTAAGCAGACAGTTTTATTGTTCATGACCAAAATCCCTTAACGTGAGTTTTCGTTCCACTGAGCGTCAGACCCCGTAGAAAAGATCAAAGGATCTTCTTGAGATCCTTTTTTTCTGCGCGTAATCTGCTGCTTGCAAACAAAAAAACCACCGCTACCAGCGGTGGTTTGTTTGCCGGATCAAGAGCTACCAACTCTTTTTCCGAAGGTAACTGGCTTCAGCAGAGCGCAGATACCAAATACTGTCCTTCTAGTGTAGCCGTAGTTAGGCCACCACTTCAAGAACTCTGTAGCACCGCCTACATACCTCGCTCTGCTAATCCTGTTACCAGTGGCTGCTGCCAGTGGCGATAAGTCGTGTCTTACCGGGTTGGACTCAAGACGATAGTTACCGGATAAGGCGCAGCGGTCGGGCTGAACGGGGGGTTCGTGCACACAGCCCAGCTTGGAGCGAACGACCTACACCGAACTGAGATACCTACAGCGTGAGCTATGAGAAAGCGCCACGCTTCCCGAAGGGAGAAAGGCGGACAGGTATCCGGTAAGCGGCAGGGTCGGAACAGGAGAGCGCACGAGGGAGCTTCCAGGGGGAAACGCCTGGTATCTTTATAGTCCTGTCGGGTTTCGCCACCTCTGACTTGAGCGTCGATTTTTGTGATGCTCGTCAGGGGGGCGGAGCCTATGGAAAAACGCCAGCAACGCGGCCTTTTTACGGTTCCTGGCCTTTTGCTGGCCTTTTGCTCACATGTTCTTTCCTGCGTTATCCCCTGATTCTGTGGATAACCGTATTACCGCCTTTGAGTGAGCTGATACCGCTCGCCGCAGCCGAACGACCGAGCGCAGCGAGTCAGTGAGCGAGGAAGCGGAAGAGCGCCTGATGCGGTATTTTCTCCTTACGCATCTGTGCGGTATTTCACACCGCATATATGGTGCACTCTCAGTACAATCTGCTCTGATGCCGCATAGTTAAGCCAGTATACACTCCGCTATCGCTACGTGACTGGGTCATGGCTGCGCCCCGACACCCGCCAACACCCGCTGACGCGCCCTGACGGGCTTGTCTGCTCCCGGCATCCGCTTACAGACAAGCTGTGACCGTCTCCGGGAGCTGCATGTGTCAGAGGTTTTCACCGTCATCACCGAAACGCGCGAGGCAGCTGCGGTAAAGCTCATCAGCGTGGTCGTGAAGCGATTCACAGATGTCTGCCTGTTCATCCGCGTCCAGCTCGTTGAGTTTCTCCAGAAGCGTTAATGTCTGGCTTCTGATAAAGCGGGCCATGTTAAGGGCGGTTTTTTCCTGTTTGGTCACTGATGCCTCCGTGTAAGGGGGATTTCTGTTCATGGGGGTAATGATACCGATGAAACGAGAGAGGATGCTCACGATACGGGTTACTGATGATGAACATGCCCGGTTACTGGAACGTTGTGAGGGTAAACAACTGGCGGTATGGATGCGGCGGGACCAGAGAAAAATCACTCAGGGTCAATGCCAGCGCTTCGTTAATACAGATGTAGGTGTTCCACAGGGTAGCCAGCAGCATCCTGCGATGCAGATCCGGAACATAATGGTGCAGGGCGCTGACTTCCGCGTTTCCAGACTTTACGAAACACGGAAACCGAAGACCATTCATGTTGTTGCTCAGGTCGCAGACGTTTTGCAGCAGCAGTCGCTTCACGTTCGCTCGCGTATCGGTGATTCATTCTGCTAACCAGTAAGGCAACCCCGCCAGCCTAGCCGGGTCCTCAACGACAGGAGCACGATCATGCGCACCCGTGGGGCCGCCATGCCGGCGATAATGGCCTGCTTCTCGCCGAAACGTTTGGTGGCGGGACCAGTGACGAAGGCTTGAGCGAGGGCGTGCAAGATTCCGAATACCGCAAGCGACAGGCCGATCATCGTCGCGCTCCAGCGAAAGCGGTCCTCGCCGAAAATGACCCAGAGCGCTGCCGGCACCTGTCCTACGAGTTGCATGATAAAGAAGACAGTCATAAGTGCGGCGACGATAGTCATGCCCCGCGCCCACCGGAAGGAGCTGACTGGGTTGAAGGCTCTCAAGGGCATCGGTCGAGATCCCGGTGCCTAATGAGTGAGCTAACTTACATTAATTGCGTTGCGCTCACTGCCCGCTTTCCAGTCGGGAAACCTGTCGTGCCAGCTGCATTAATGAATCGGCCAACGCGCGGGGAGAGGCGGTTTGCGTATTGGGCGCCAGGGTGGTTTTTCTTTTCACCAGTGAGACGGGCAACAGCTGATTGCCCTTCACCGCCTGGCCCTGAGAGAGTTGCAGCAAGCGGTCCACGCTGGTTTGCCCCAGCAGGCGAAAATCCTGTTTGATGGTGGTTAACGGCGGGATATAACATGAGCTGTCTTCGGTATCGTCGTATCCCACTACCGAGATATCCGCACCAACGCGCAGCCCGGACTCGGTAATGGCGCGCATTGCGCCCAGCGCCATCTGATCGTTGGCAACCAGCATCGCAGTGGGAACGATGCCCTCATTCAGCATTTGCATGGTTTGTTGAAAACCGGACATGGCACTCCAGTCGCCTTCCCGTTCCGCTATCGGCTGAATTTGATTGCGAGTGAGATATTTATGCCAGCCAGCCAGACGCAGACGCGCCGAGACAGAACTTAATGGGCCCGCTAACAGCGCGATTTGCTGGTGACCCAATGCGACCAGATGCTCCACGCCCAGTCGCGTACCGTCTTCATGGGAGAAAATAATACTGTTGATGGGTGTCTGGTCAGAGACATCAAGAAATAACGCCGGAACATTAGTGCAGGCAGCTTCCACAGCAATGGCATCCTGGTCATCCAGCGGATAGTTAATGATCAGCCCACTGACGCGTTGCGCGAGAAGATTGTGCACCGCCGCTTTACAGGCTTCGACGCCGCTTCGTTCTACCATCGACACCACCACGCTGGCACCCAGTTGATCGGCGCGAGATTTAATCGCCGCGACAATTTGCGACGGCGCGTGCAGGGCCAGACTGGAGGTGGCAACGCCAATCAGCAACGACTGTTTGCCCGCCAGTTGTTGTGCCACGCGGTTGGGAATGTAATTCAGCTCCGCCATCGCCGCTTCCACTTTTTCCCGCGTTTTCGCAGAAACGTGGCTGGCCTGGTTCACCACGCGGGAAACGGTCTGATAAGAGACACCGGCATACTCTGCGACATCGTATAACGTTACTGGTTTCACATTCACCACCCTGAATTGACTCTCTTCCGGGCGCTATCATGCCATACCGCGAAAGGTTTTGCGCCATTCGATGGTGTCCGGGATCTCGACGCTCTCCCTTATGCGACTCCTGCATTAGGAAGCAGCCCAGTAGTAGGTTGAGGCCGTTGAGCACCGCCGCCGCAAGGAATGGTGCATGCAAGGAGATGGCGCCCAACAGTCCCCCGGCCACGGGGCCTGCCACCATACCCACGCCGAAACAAGCGCTCATGAGCCCGAAGTGGCGAGCCCGATCTTCCCCATCGGTGATGTCGGCGATATAGGCGCCAGCAACCGCACCTGTGGCGCCGGTGATGCCGGCCACGATGCGTCCGGCGTAGAGGATCGAGATCTCGATCCCGCGAAATTAATACGACTCACTATAGGGGAATTGTGAGCGGATAACAATTCCCCTCTAGAAATAATTTTGTTTAACTTTAAGAAGGAGATATACCATGAAATACCTATTGCCTACGGCAGCCGCTGGATTGTTATTACTCGCGGCCCAGCCGGCCATGGCATCTAGGACTCCGGAAATGCCGGTTCTGGAAAATCGTGCAGCACAGGGTGATATTACCGCACCGGGTGGTGCACGTCGTCTGACCGGTGATCAGACCGCAGCACTGCGTGATAGCCTGAGCGATAAACCGGCAAAAAACATTATTCTGCTGATTGGTGATGGCATGGGTGATAGCGAAATTACCGCAGCACGTAATTATGCCGAAGGTGCCGGTGGTTTTTTTAAAGGTATTGATGCACTGCCGCTGACAGGTCAGTATACCCATTATGCACTGAATAAAAAAACCGGCAAACCGGATTATGTTACCGATAGCGCAGCAAGCGCAACCGCATGGTCAACCGGTGTTAAAACCTATAATGGTGCACTGGGTGTGGATATTCATGAAAAAGATCATCCGACCATTCTGGAAATGGCAAAAGCAGCAGGTCTGGCAACCGGTAATGTTAGCACCGCAGAACTGCAGGATGCAACACCGGCAGCACTGGTTGCACATGTTACCAGCCGTAAATGTTATGGTCCGAGCGCAACCAGCGAAAAATGTCCGGGTAATGCACTGGAAAAAGGTGGTAAAGGTAGCATTACCGAACAGCTGCTGAATGCACGTGCAGATGTTACCCTGGGTGGTGGTGCAAAAACCTTTGCAGAAACCGCAACCGCAGGCGAATGGCAGGGTAAAACCCTGCGTGAACAGGCACAGGCACGTGGTTATCAGCTGGTTAGTGATGCAGCAAGCCTGAATAGCGTTACCGAAGCAAATCAGCAGAAACCGCTGCTGGGTCTGTTTGCAGATGGTAATATGCCGGTTCGTTGGCTGGGTCCGAAAGCAACCTATCATGGTAATATTGATAAACCGGCTGTTACCTGTACCCCGAATCCGCAGCGTAATGATAGCGTTCCGACCCTGGCACAGATGACCGATAAAGCAATTGAACTGCTGAGCAAAAATGAAAAAGGCTTTTTTCTGCAGGTTGAAGGTGCCAGCATTGATAAACAGGATCATGCAGCAAATCCGTGTGGTCAGATTGGTGAAACCGTTGATCTGGATGAAGCAGTTCAGCGTGCACTGGAATTTGCAAAAAAAGAAGGTAATACCCTGGTTATTGTGACCGCAGATCATGCACATGCAAGCCAGATTGTTGCACCGGATACCAAAGCACCGGGTCTGACCCAGGCACTGAATACCAAAGATGGTGCAGTTATGGTTATGAGCTATGGTAATAGCGAAGAAGATAGCCAGGAACATACCGGTAGCCAGCTGCGTATTGCAGCATATGGTCCGCATGCAGCCAATGTTGTTGGTCTGACCGATCAAACCGACCTGTTTTATACCATGAAAGCAGCACTGGGTCTGAAAGGCGGCGGTGGTAGCAAAGGTGGTGGCTCTGTGGCCGAGGCGGAAGCGTGGGAACAGTGCGGTGGATCAGACTGGACTGGTCCCAAACAGTGTCCTATGGATCACACGTGTCTGGTGCGGCGTGAAAAATTCAGCCAGTGTGTACCAGTTTGGTTGGATGATTGGGGAGGTTCAAAGGGCGAGCACCACCACCACCACCACTGAGATCCGGCTGCTAACAAAGCCCGAAAGGAAGCTGAGTTGGCTGCTGCCACCGCTGAGCAATAACTAGCATAACCCCTTGGGGCCTCTAAACGGGTCTTGAGGGGTTTTTTGCTGAAAGGAGGAACTATATCCGGAT

**pBR3h pelB-AP-EHUX1b**

TGGCGAATGGGACGCGCCCTGTAGCGGCGCATTAAGCGCGGCGGGTGTGGTGGTTACGCGCAGCGTGACCGCTACACTTGCCAGCGCCCTAGCGCCCGCTCCTTTCGCTTTCTTCCCTTCCTTTCTCGCCACGTTCGCCGGCTTTCCCCGTCAAGCTCTAAATCGGGGGCTCCCTTTAGGGTTCCGATTTAGTGCTTTACGGCACCTCGACCCCAAAAAACTTGATTAGGGTGATGGTTCACGTAGTGGGCCATCGCCCTGATAGACGGTTTTTCGCCCTTTGACGTTGGAGTCCACGTTCTTTAATAGTGGACTCTTGTTCCAAACTGGAACAACACTCAACCCTATCTCGGTCTATTCTTTTGATTTATAAGGGATTTTGCCGATTTCGGCCTATTGGTTAAAAAATGAGCTGATTTAACAAAAATTTAACGCGAATTTTAACAAAATATTAACGCTTACAATTTAGGTGGCACTTTTCGGGGAAATGTGCGCGGAACCCCTATTTGTTTATTTTTCTAAATACATTCAAATATGTATCCGCTCATGAATTAATTCTTAGAAAAACTCATCGAGCATCAAATGAAACTGCAATTTATTCATATCAGGATTATCAATACCATATTTTTGAAAAAGCCGTTTCTGTAATGAAGGAGAAAACTCACCGAGGCAGTTCCATAGGATGGCAAGATCCTGGTATCGGTCTGCGATTCCGACTCGTCCAACATCAATACAACCTATTAATTTCCCCTCGTCAAAAATAAGGTTATCAAGTGAGAAATCACCATGAGTGACGACTGAATCCGGTGAGAATGGCAAAAGTTTATGCATTTCTTTCCAGACTTGTTCAACAGGCCAGCCATTACGCTCGTCATCAAAATCACTCGCATCAACCAAACCGTTATTCATTCGTGATTGCGCCTGAGCGAGACGAAATACGCGATCGCTGTTAAAAGGACAATTACAAACAGGAATCGAATGCAACCGGCGCAGGAACACTGCCAGCGCATCAACAATATTTTCACCTGAATCAGGATATTCTTCTAATACCTGGAATGCTGTTTTCCCGGGGATCGCAGTGGTGAGTAACCATGCATCATCAGGAGTACGGATAAAATGCTTGATGGTCGGAAGAGGCATAAATTCCGTCAGCCAGTTTAGTCTGACCATCTCATCTGTAACATCATTGGCAACGCTACCTTTGCCATGTTTCAGAAACAACTCTGGCGCATCGGGCTTCCCATACAATCGATAGATTGTCGCACCTGATTGCCCGACATTATCGCGAGCCCATTTATACCCATATAAATCAGCATCCATGTTGGAATTTAATCGCGGCCTAGAGCAAGACGTTTCCCGTTGAATATGGCTCATAACACCCCTTGTATTACTGTTTATGTAAGCAGACAGTTTTATTGTTCATGACCAAAATCCCTTAACGTGAGTTTTCGTTCCACTGAGCGTCAGACCCCGTAGAAAAGATCAAAGGATCTTCTTGAGATCCTTTTTTTCTGCGCGTAATCTGCTGCTTGCAAACAAAAAAACCACCGCTACCAGCGGTGGTTTGTTTGCCGGATCAAGAGCTACCAACTCTTTTTCCGAAGGTAACTGGCTTCAGCAGAGCGCAGATACCAAATACTGTCCTTCTAGTGTAGCCGTAGTTAGGCCACCACTTCAAGAACTCTGTAGCACCGCCTACATACCTCGCTCTGCTAATCCTGTTACCAGTGGCTGCTGCCAGTGGCGATAAGTCGTGTCTTACCGGGTTGGACTCAAGACGATAGTTACCGGATAAGGCGCAGCGGTCGGGCTGAACGGGGGGTTCGTGCACACAGCCCAGCTTGGAGCGAACGACCTACACCGAACTGAGATACCTACAGCGTGAGCTATGAGAAAGCGCCACGCTTCCCGAAGGGAGAAAGGCGGACAGGTATCCGGTAAGCGGCAGGGTCGGAACAGGAGAGCGCACGAGGGAGCTTCCAGGGGGAAACGCCTGGTATCTTTATAGTCCTGTCGGGTTTCGCCACCTCTGACTTGAGCGTCGATTTTTGTGATGCTCGTCAGGGGGGCGGAGCCTATGGAAAAACGCCAGCAACGCGGCCTTTTTACGGTTCCTGGCCTTTTGCTGGCCTTTTGCTCACATGTTCTTTCCTGCGTTATCCCCTGATTCTGTGGATAACCGTATTACCGCCTTTGAGTGAGCTGATACCGCTCGCCGCAGCCGAACGACCGAGCGCAGCGAGTCAGTGAGCGAGGAAGCGGAAGAGCGCCTGATGCGGTATTTTCTCCTTACGCATCTGTGCGGTATTTCACACCGCATATATGGTGCACTCTCAGTACAATCTGCTCTGATGCCGCATAGTTAAGCCAGTATACACTCCGCTATCGCTACGTGACTGGGTCATGGCTGCGCCCCGACACCCGCCAACACCCGCTGACGCGCCCTGACGGGCTTGTCTGCTCCCGGCATCCGCTTACAGACAAGCTGTGACCGTCTCCGGGAGCTGCATGTGTCAGAGGTTTTCACCGTCATCACCGAAACGCGCGAGGCAGCTGCGGTAAAGCTCATCAGCGTGGTCGTGAAGCGATTCACAGATGTCTGCCTGTTCATCCGCGTCCAGCTCGTTGAGTTTCTCCAGAAGCGTTAATGTCTGGCTTCTGATAAAGCGGGCCATGTTAAGGGCGGTTTTTTCCTGTTTGGTCACTGATGCCTCCGTGTAAGGGGGATTTCTGTTCATGGGGGTAATGATACCGATGAAACGAGAGAGGATGCTCACGATACGGGTTACTGATGATGAACATGCCCGGTTACTGGAACGTTGTGAGGGTAAACAACTGGCGGTATGGATGCGGCGGGACCAGAGAAAAATCACTCAGGGTCAATGCCAGCGCTTCGTTAATACAGATGTAGGTGTTCCACAGGGTAGCCAGCAGCATCCTGCGATGCAGATCCGGAACATAATGGTGCAGGGCGCTGACTTCCGCGTTTCCAGACTTTACGAAACACGGAAACCGAAGACCATTCATGTTGTTGCTCAGGTCGCAGACGTTTTGCAGCAGCAGTCGCTTCACGTTCGCTCGCGTATCGGTGATTCATTCTGCTAACCAGTAAGGCAACCCCGCCAGCCTAGCCGGGTCCTCAACGACAGGAGCACGATCATGCGCACCCGTGGGGCCGCCATGCCGGCGATAATGGCCTGCTTCTCGCCGAAACGTTTGGTGGCGGGACCAGTGACGAAGGCTTGAGCGAGGGCGTGCAAGATTCCGAATACCGCAAGCGACAGGCCGATCATCGTCGCGCTCCAGCGAAAGCGGTCCTCGCCGAAAATGACCCAGAGCGCTGCCGGCACCTGTCCTACGAGTTGCATGATAAAGAAGACAGTCATAAGTGCGGCGACGATAGTCATGCCCCGCGCCCACCGGAAGGAGCTGACTGGGTTGAAGGCTCTCAAGGGCATCGGTCGAGATCCCGGTGCCTAATGAGTGAGCTAACTTACATTAATTGCGTTGCGCTCACTGCCCGCTTTCCAGTCGGGAAACCTGTCGTGCCAGCTGCATTAATGAATCGGCCAACGCGCGGGGAGAGGCGGTTTGCGTATTGGGCGCCAGGGTGGTTTTTCTTTTCACCAGTGAGACGGGCAACAGCTGATTGCCCTTCACCGCCTGGCCCTGAGAGAGTTGCAGCAAGCGGTCCACGCTGGTTTGCCCCAGCAGGCGAAAATCCTGTTTGATGGTGGTTAACGGCGGGATATAACATGAGCTGTCTTCGGTATCGTCGTATCCCACTACCGAGATATCCGCACCAACGCGCAGCCCGGACTCGGTAATGGCGCGCATTGCGCCCAGCGCCATCTGATCGTTGGCAACCAGCATCGCAGTGGGAACGATGCCCTCATTCAGCATTTGCATGGTTTGTTGAAAACCGGACATGGCACTCCAGTCGCCTTCCCGTTCCGCTATCGGCTGAATTTGATTGCGAGTGAGATATTTATGCCAGCCAGCCAGACGCAGACGCGCCGAGACAGAACTTAATGGGCCCGCTAACAGCGCGATTTGCTGGTGACCCAATGCGACCAGATGCTCCACGCCCAGTCGCGTACCGTCTTCATGGGAGAAAATAATACTGTTGATGGGTGTCTGGTCAGAGACATCAAGAAATAACGCCGGAACATTAGTGCAGGCAGCTTCCACAGCAATGGCATCCTGGTCATCCAGCGGATAGTTAATGATCAGCCCACTGACGCGTTGCGCGAGAAGATTGTGCACCGCCGCTTTACAGGCTTCGACGCCGCTTCGTTCTACCATCGACACCACCACGCTGGCACCCAGTTGATCGGCGCGAGATTTAATCGCCGCGACAATTTGCGACGGCGCGTGCAGGGCCAGACTGGAGGTGGCAACGCCAATCAGCAACGACTGTTTGCCCGCCAGTTGTTGTGCCACGCGGTTGGGAATGTAATTCAGCTCCGCCATCGCCGCTTCCACTTTTTCCCGCGTTTTCGCAGAAACGTGGCTGGCCTGGTTCACCACGCGGGAAACGGTCTGATAAGAGACACCGGCATACTCTGCGACATCGTATAACGTTACTGGTTTCACATTCACCACCCTGAATTGACTCTCTTCCGGGCGCTATCATGCCATACCGCGAAAGGTTTTGCGCCATTCGATGGTGTCCGGGATCTCGACGCTCTCCCTTATGCGACTCCTGCATTAGGAAGCAGCCCAGTAGTAGGTTGAGGCCGTTGAGCACCGCCGCCGCAAGGAATGGTGCATGCAAGGAGATGGCGCCCAACAGTCCCCCGGCCACGGGGCCTGCCACCATACCCACGCCGAAACAAGCGCTCATGAGCCCGAAGTGGCGAGCCCGATCTTCCCCATCGGTGATGTCGGCGATATAGGCGCCAGCAACCGCACCTGTGGCGCCGGTGATGCCGGCCACGATGCGTCCGGCGTAGAGGATCGAGATCTCGATCCCGCGAAATTAATACGACTCACTATAGGGGAATTGTGAGCGGATAACAATTCCCCTCTAGAAATAATTTTGTTTAACTTTAAGAAGGAGATATACCATGAAATACCTATTGCCTACGGCAGCCGCTGGATTGTTATTACTCGCGGCCCAGCCGGCCATGGCATCTAGGACTCCGGAAATGCCGGTTCTGGAAAATCGTGCAGCACAGGGTGATATTACCGCACCGGGTGGTGCACGTCGTCTGACCGGTGATCAGACCGCAGCACTGCGTGATAGCCTGAGCGATAAACCGGCAAAAAACATTATTCTGCTGATTGGTGATGGCATGGGTGATAGCGAAATTACCGCAGCACGTAATTATGCCGAAGGTGCCGGTGGTTTTTTTAAAGGTATTGATGCACTGCCGCTGACAGGTCAGTATACCCATTATGCACTGAATAAAAAAACCGGCAAACCGGATTATGTTACCGATAGCGCAGCAAGCGCAACCGCATGGTCAACCGGTGTTAAAACCTATAATGGTGCACTGGGTGTGGATATTCATGAAAAAGATCATCCGACCATTCTGGAAATGGCAAAAGCAGCAGGTCTGGCAACCGGTAATGTTAGCACCGCAGAACTGCAGGATGCAACACCGGCAGCACTGGTTGCACATGTTACCAGCCGTAAATGTTATGGTCCGAGCGCAACCAGCGAAAAATGTCCGGGTAATGCACTGGAAAAAGGTGGTAAAGGTAGCATTACCGAACAGCTGCTGAATGCACGTGCAGATGTTACCCTGGGTGGTGGTGCAAAAACCTTTGCAGAAACCGCAACCGCAGGCGAATGGCAGGGTAAAACCCTGCGTGAACAGGCACAGGCACGTGGTTATCAGCTGGTTAGTGATGCAGCAAGCCTGAATAGCGTTACCGAAGCAAATCAGCAGAAACCGCTGCTGGGTCTGTTTGCAGATGGTAATATGCCGGTTCGTTGGCTGGGTCCGAAAGCAACCTATCATGGTAATATTGATAAACCGGCTGTTACCTGTACCCCGAATCCGCAGCGTAATGATAGCGTTCCGACCCTGGCACAGATGACCGATAAAGCAATTGAACTGCTGAGCAAAAATGAAAAAGGCTTTTTTCTGCAGGTTGAAGGTGCCAGCATTGATAAACAGGATCATGCAGCAAATCCGTGTGGTCAGATTGGTGAAACCGTTGATCTGGATGAAGCAGTTCAGCGTGCACTGGAATTTGCAAAAAAAGAAGGTAATACCCTGGTTATTGTGACCGCAGATCATGCACATGCAAGCCAGATTGTTGCACCGGATACCAAAGCACCGGGTCTGACCCAGGCACTGAATACCAAAGATGGTGCAGTTATGGTTATGAGCTATGGTAATAGCGAAGAAGATAGCCAGGAACATACCGGTAGCCAGCTGCGTATTGCAGCATATGGTCCGCATGCAGCCAATGTTGTTGGTCTGACCGATCAAACCGACCTGTTTTATACCATGAAAGCAGCACTGGGTCTGAAAGGCGGCGGTGGTAGCAAAGGTGGTGGCTCTCCACGTAACCCCGGTCCTTGGGAACAATGCGGCGGAAAATCGTACGAAGGTCCGACGGCCTGTCCCCGTGAATACACGTGTCAGTATCGCCGTGAAACGTTTTCTCAGTGTATTCCACAGTTTATTGCCAAATGGGGAGGTTCAAAGGGCGAGCACCACCACCACCACCACTGAGATCCGGCTGCTAACAAAGCCCGAAAGGAAGCTGAGTTGGCTGCTGCCACCGCTGAGCAATAACTAGCATAACCCCTTGGGGCCTCTAAACGGGTCTTGAGGGGTTTTTTGCTGAAAGGAGGAACTATATCCGGAT

**pBR3i pelB-AP-EHUX2**

TGGCGAATGGGACGCGCCCTGTAGCGGCGCATTAAGCGCGGCGGGTGTGGTGGTTACGCGCAGCGTGACCGCTACACTTGCCAGCGCCCTAGCGCCCGCTCCTTTCGCTTTCTTCCCTTCCTTTCTCGCCACGTTCGCCGGCTTTCCCCGTCAAGCTCTAAATCGGGGGCTCCCTTTAGGGTTCCGATTTAGTGCTTTACGGCACCTCGACCCCAAAAAACTTGATTAGGGTGATGGTTCACGTAGTGGGCCATCGCCCTGATAGACGGTTTTTCGCCCTTTGACGTTGGAGTCCACGTTCTTTAATAGTGGACTCTTGTTCCAAACTGGAACAACACTCAACCCTATCTCGGTCTATTCTTTTGATTTATAAGGGATTTTGCCGATTTCGGCCTATTGGTTAAAAAATGAGCTGATTTAACAAAAATTTAACGCGAATTTTAACAAAATATTAACGCTTACAATTTAGGTGGCACTTTTCGGGGAAATGTGCGCGGAACCCCTATTTGTTTATTTTTCTAAATACATTCAAATATGTATCCGCTCATGAATTAATTCTTAGAAAAACTCATCGAGCATCAAATGAAACTGCAATTTATTCATATCAGGATTATCAATACCATATTTTTGAAAAAGCCGTTTCTGTAATGAAGGAGAAAACTCACCGAGGCAGTTCCATAGGATGGCAAGATCCTGGTATCGGTCTGCGATTCCGACTCGTCCAACATCAATACAACCTATTAATTTCCCCTCGTCAAAAATAAGGTTATCAAGTGAGAAATCACCATGAGTGACGACTGAATCCGGTGAGAATGGCAAAAGTTTATGCATTTCTTTCCAGACTTGTTCAACAGGCCAGCCATTACGCTCGTCATCAAAATCACTCGCATCAACCAAACCGTTATTCATTCGTGATTGCGCCTGAGCGAGACGAAATACGCGATCGCTGTTAAAAGGACAATTACAAACAGGAATCGAATGCAACCGGCGCAGGAACACTGCCAGCGCATCAACAATATTTTCACCTGAATCAGGATATTCTTCTAATACCTGGAATGCTGTTTTCCCGGGGATCGCAGTGGTGAGTAACCATGCATCATCAGGAGTACGGATAAAATGCTTGATGGTCGGAAGAGGCATAAATTCCGTCAGCCAGTTTAGTCTGACCATCTCATCTGTAACATCATTGGCAACGCTACCTTTGCCATGTTTCAGAAACAACTCTGGCGCATCGGGCTTCCCATACAATCGATAGATTGTCGCACCTGATTGCCCGACATTATCGCGAGCCCATTTATACCCATATAAATCAGCATCCATGTTGGAATTTAATCGCGGCCTAGAGCAAGACGTTTCCCGTTGAATATGGCTCATAACACCCCTTGTATTACTGTTTATGTAAGCAGACAGTTTTATTGTTCATGACCAAAATCCCTTAACGTGAGTTTTCGTTCCACTGAGCGTCAGACCCCGTAGAAAAGATCAAAGGATCTTCTTGAGATCCTTTTTTTCTGCGCGTAATCTGCTGCTTGCAAACAAAAAAACCACCGCTACCAGCGGTGGTTTGTTTGCCGGATCAAGAGCTACCAACTCTTTTTCCGAAGGTAACTGGCTTCAGCAGAGCGCAGATACCAAATACTGTCCTTCTAGTGTAGCCGTAGTTAGGCCACCACTTCAAGAACTCTGTAGCACCGCCTACATACCTCGCTCTGCTAATCCTGTTACCAGTGGCTGCTGCCAGTGGCGATAAGTCGTGTCTTACCGGGTTGGACTCAAGACGATAGTTACCGGATAAGGCGCAGCGGTCGGGCTGAACGGGGGGTTCGTGCACACAGCCCAGCTTGGAGCGAACGACCTACACCGAACTGAGATACCTACAGCGTGAGCTATGAGAAAGCGCCACGCTTCCCGAAGGGAGAAAGGCGGACAGGTATCCGGTAAGCGGCAGGGTCGGAACAGGAGAGCGCACGAGGGAGCTTCCAGGGGGAAACGCCTGGTATCTTTATAGTCCTGTCGGGTTTCGCCACCTCTGACTTGAGCGTCGATTTTTGTGATGCTCGTCAGGGGGGCGGAGCCTATGGAAAAACGCCAGCAACGCGGCCTTTTTACGGTTCCTGGCCTTTTGCTGGCCTTTTGCTCACATGTTCTTTCCTGCGTTATCCCCTGATTCTGTGGATAACCGTATTACCGCCTTTGAGTGAGCTGATACCGCTCGCCGCAGCCGAACGACCGAGCGCAGCGAGTCAGTGAGCGAGGAAGCGGAAGAGCGCCTGATGCGGTATTTTCTCCTTACGCATCTGTGCGGTATTTCACACCGCATATATGGTGCACTCTCAGTACAATCTGCTCTGATGCCGCATAGTTAAGCCAGTATACACTCCGCTATCGCTACGTGACTGGGTCATGGCTGCGCCCCGACACCCGCCAACACCCGCTGACGCGCCCTGACGGGCTTGTCTGCTCCCGGCATCCGCTTACAGACAAGCTGTGACCGTCTCCGGGAGCTGCATGTGTCAGAGGTTTTCACCGTCATCACCGAAACGCGCGAGGCAGCTGCGGTAAAGCTCATCAGCGTGGTCGTGAAGCGATTCACAGATGTCTGCCTGTTCATCCGCGTCCAGCTCGTTGAGTTTCTCCAGAAGCGTTAATGTCTGGCTTCTGATAAAGCGGGCCATGTTAAGGGCGGTTTTTTCCTGTTTGGTCACTGATGCCTCCGTGTAAGGGGGATTTCTGTTCATGGGGGTAATGATACCGATGAAACGAGAGAGGATGCTCACGATACGGGTTACTGATGATGAACATGCCCGGTTACTGGAACGTTGTGAGGGTAAACAACTGGCGGTATGGATGCGGCGGGACCAGAGAAAAATCACTCAGGGTCAATGCCAGCGCTTCGTTAATACAGATGTAGGTGTTCCACAGGGTAGCCAGCAGCATCCTGCGATGCAGATCCGGAACATAATGGTGCAGGGCGCTGACTTCCGCGTTTCCAGACTTTACGAAACACGGAAACCGAAGACCATTCATGTTGTTGCTCAGGTCGCAGACGTTTTGCAGCAGCAGTCGCTTCACGTTCGCTCGCGTATCGGTGATTCATTCTGCTAACCAGTAAGGCAACCCCGCCAGCCTAGCCGGGTCCTCAACGACAGGAGCACGATCATGCGCACCCGTGGGGCCGCCATGCCGGCGATAATGGCCTGCTTCTCGCCGAAACGTTTGGTGGCGGGACCAGTGACGAAGGCTTGAGCGAGGGCGTGCAAGATTCCGAATACCGCAAGCGACAGGCCGATCATCGTCGCGCTCCAGCGAAAGCGGTCCTCGCCGAAAATGACCCAGAGCGCTGCCGGCACCTGTCCTACGAGTTGCATGATAAAGAAGACAGTCATAAGTGCGGCGACGATAGTCATGCCCCGCGCCCACCGGAAGGAGCTGACTGGGTTGAAGGCTCTCAAGGGCATCGGTCGAGATCCCGGTGCCTAATGAGTGAGCTAACTTACATTAATTGCGTTGCGCTCACTGCCCGCTTTCCAGTCGGGAAACCTGTCGTGCCAGCTGCATTAATGAATCGGCCAACGCGCGGGGAGAGGCGGTTTGCGTATTGGGCGCCAGGGTGGTTTTTCTTTTCACCAGTGAGACGGGCAACAGCTGATTGCCCTTCACCGCCTGGCCCTGAGAGAGTTGCAGCAAGCGGTCCACGCTGGTTTGCCCCAGCAGGCGAAAATCCTGTTTGATGGTGGTTAACGGCGGGATATAACATGAGCTGTCTTCGGTATCGTCGTATCCCACTACCGAGATATCCGCACCAACGCGCAGCCCGGACTCGGTAATGGCGCGCATTGCGCCCAGCGCCATCTGATCGTTGGCAACCAGCATCGCAGTGGGAACGATGCCCTCATTCAGCATTTGCATGGTTTGTTGAAAACCGGACATGGCACTCCAGTCGCCTTCCCGTTCCGCTATCGGCTGAATTTGATTGCGAGTGAGATATTTATGCCAGCCAGCCAGACGCAGACGCGCCGAGACAGAACTTAATGGGCCCGCTAACAGCGCGATTTGCTGGTGACCCAATGCGACCAGATGCTCCACGCCCAGTCGCGTACCGTCTTCATGGGAGAAAATAATACTGTTGATGGGTGTCTGGTCAGAGACATCAAGAAATAACGCCGGAACATTAGTGCAGGCAGCTTCCACAGCAATGGCATCCTGGTCATCCAGCGGATAGTTAATGATCAGCCCACTGACGCGTTGCGCGAGAAGATTGTGCACCGCCGCTTTACAGGCTTCGACGCCGCTTCGTTCTACCATCGACACCACCACGCTGGCACCCAGTTGATCGGCGCGAGATTTAATCGCCGCGACAATTTGCGACGGCGCGTGCAGGGCCAGACTGGAGGTGGCAACGCCAATCAGCAACGACTGTTTGCCCGCCAGTTGTTGTGCCACGCGGTTGGGAATGTAATTCAGCTCCGCCATCGCCGCTTCCACTTTTTCCCGCGTTTTCGCAGAAACGTGGCTGGCCTGGTTCACCACGCGGGAAACGGTCTGATAAGAGACACCGGCATACTCTGCGACATCGTATAACGTTACTGGTTTCACATTCACCACCCTGAATTGACTCTCTTCCGGGCGCTATCATGCCATACCGCGAAAGGTTTTGCGCCATTCGATGGTGTCCGGGATCTCGACGCTCTCCCTTATGCGACTCCTGCATTAGGAAGCAGCCCAGTAGTAGGTTGAGGCCGTTGAGCACCGCCGCCGCAAGGAATGGTGCATGCAAGGAGATGGCGCCCAACAGTCCCCCGGCCACGGGGCCTGCCACCATACCCACGCCGAAACAAGCGCTCATGAGCCCGAAGTGGCGAGCCCGATCTTCCCCATCGGTGATGTCGGCGATATAGGCGCCAGCAACCGCACCTGTGGCGCCGGTGATGCCGGCCACGATGCGTCCGGCGTAGAGGATCGAGATCTCGATCCCGCGAAATTAATACGACTCACTATAGGGGAATTGTGAGCGGATAACAATTCCCCTCTAGAAATAATTTTGTTTAACTTTAAGAAGGAGATATACCATGAAATACCTATTGCCTACGGCAGCCGCTGGATTGTTATTACTCGCGGCCCAGCCGGCCATGGCATCTAGGACTCCGGAAATGCCGGTTCTGGAAAATCGTGCAGCACAGGGTGATATTACCGCACCGGGTGGTGCACGTCGTCTGACCGGTGATCAGACCGCAGCACTGCGTGATAGCCTGAGCGATAAACCGGCAAAAAACATTATTCTGCTGATTGGTGATGGCATGGGTGATAGCGAAATTACCGCAGCACGTAATTATGCCGAAGGTGCCGGTGGTTTTTTTAAAGGTATTGATGCACTGCCGCTGACAGGTCAGTATACCCATTATGCACTGAATAAAAAAACCGGCAAACCGGATTATGTTACCGATAGCGCAGCAAGCGCAACCGCATGGTCAACCGGTGTTAAAACCTATAATGGTGCACTGGGTGTGGATATTCATGAAAAAGATCATCCGACCATTCTGGAAATGGCAAAAGCAGCAGGTCTGGCAACCGGTAATGTTAGCACCGCAGAACTGCAGGATGCAACACCGGCAGCACTGGTTGCACATGTTACCAGCCGTAAATGTTATGGTCCGAGCGCAACCAGCGAAAAATGTCCGGGTAATGCACTGGAAAAAGGTGGTAAAGGTAGCATTACCGAACAGCTGCTGAATGCACGTGCAGATGTTACCCTGGGTGGTGGTGCAAAAACCTTTGCAGAAACCGCAACCGCAGGCGAATGGCAGGGTAAAACCCTGCGTGAACAGGCACAGGCACGTGGTTATCAGCTGGTTAGTGATGCAGCAAGCCTGAATAGCGTTACCGAAGCAAATCAGCAGAAACCGCTGCTGGGTCTGTTTGCAGATGGTAATATGCCGGTTCGTTGGCTGGGTCCGAAAGCAACCTATCATGGTAATATTGATAAACCGGCTGTTACCTGTACCCCGAATCCGCAGCGTAATGATAGCGTTCCGACCCTGGCACAGATGACCGATAAAGCAATTGAACTGCTGAGCAAAAATGAAAAAGGCTTTTTTCTGCAGGTTGAAGGTGCCAGCATTGATAAACAGGATCATGCAGCAAATCCGTGTGGTCAGATTGGTGAAACCGTTGATCTGGATGAAGCAGTTCAGCGTGCACTGGAATTTGCAAAAAAAGAAGGTAATACCCTGGTTATTGTGACCGCAGATCATGCACATGCAAGCCAGATTGTTGCACCGGATACCAAAGCACCGGGTCTGACCCAGGCACTGAATACCAAAGATGGTGCAGTTATGGTTATGAGCTATGGTAATAGCGAAGAAGATAGCCAGGAACATACCGGTAGCCAGCTGCGTATTGCAGCATATGGTCCGCATGCAGCCAATGTTGTTGGTCTGACCGATCAAACCGACCTGTTTTATACCATGAAAGCAGCACTGGGTCTGAAAGGCGGCGGTGGTAGCAAAGGTGGTGGCTCTACGATCTGCCAGCTGAAATGGGCGCAATGTGGCGGGAACTCATGGACGGGCGCAACTTGCTGTGAAGCCGGATCTGTATGTACGGCAGACAGTGCCTGGTACAGTCAGTGTAAACCAAGCCGCAATTACTTAGGAGGTTCAAAGGGCGAGCACCACCACCACCACCACTGAGATCCGGCTGCTAACAAAGCCCGAAAGGAAGCTGAGTTGGCTGCTGCCACCGCTGAGCAATAACTAGCATAACCCCTTGGGGCCTCTAAACGGGTCTTGAGGGGTTTTTTGCTGAAAGGAGGAACTATATCCGGAT

**pBR3j pelB-AP-EHUX3**

TGGCGAATGGGACGCGCCCTGTAGCGGCGCATTAAGCGCGGCGGGTGTGGTGGTTACGCGCAGCGTGACCGCTACACTTGCCAGCGCCCTAGCGCCCGCTCCTTTCGCTTTCTTCCCTTCCTTTCTCGCCACGTTCGCCGGCTTTCCCCGTCAAGCTCTAAATCGGGGGCTCCCTTTAGGGTTCCGATTTAGTGCTTTACGGCACCTCGACCCCAAAAAACTTGATTAGGGTGATGGTTCACGTAGTGGGCCATCGCCCTGATAGACGGTTTTTCGCCCTTTGACGTTGGAGTCCACGTTCTTTAATAGTGGACTCTTGTTCCAAACTGGAACAACACTCAACCCTATCTCGGTCTATTCTTTTGATTTATAAGGGATTTTGCCGATTTCGGCCTATTGGTTAAAAAATGAGCTGATTTAACAAAAATTTAACGCGAATTTTAACAAAATATTAACGCTTACAATTTAGGTGGCACTTTTCGGGGAAATGTGCGCGGAACCCCTATTTGTTTATTTTTCTAAATACATTCAAATATGTATCCGCTCATGAATTAATTCTTAGAAAAACTCATCGAGCATCAAATGAAACTGCAATTTATTCATATCAGGATTATCAATACCATATTTTTGAAAAAGCCGTTTCTGTAATGAAGGAGAAAACTCACCGAGGCAGTTCCATAGGATGGCAAGATCCTGGTATCGGTCTGCGATTCCGACTCGTCCAACATCAATACAACCTATTAATTTCCCCTCGTCAAAAATAAGGTTATCAAGTGAGAAATCACCATGAGTGACGACTGAATCCGGTGAGAATGGCAAAAGTTTATGCATTTCTTTCCAGACTTGTTCAACAGGCCAGCCATTACGCTCGTCATCAAAATCACTCGCATCAACCAAACCGTTATTCATTCGTGATTGCGCCTGAGCGAGACGAAATACGCGATCGCTGTTAAAAGGACAATTACAAACAGGAATCGAATGCAACCGGCGCAGGAACACTGCCAGCGCATCAACAATATTTTCACCTGAATCAGGATATTCTTCTAATACCTGGAATGCTGTTTTCCCGGGGATCGCAGTGGTGAGTAACCATGCATCATCAGGAGTACGGATAAAATGCTTGATGGTCGGAAGAGGCATAAATTCCGTCAGCCAGTTTAGTCTGACCATCTCATCTGTAACATCATTGGCAACGCTACCTTTGCCATGTTTCAGAAACAACTCTGGCGCATCGGGCTTCCCATACAATCGATAGATTGTCGCACCTGATTGCCCGACATTATCGCGAGCCCATTTATACCCATATAAATCAGCATCCATGTTGGAATTTAATCGCGGCCTAGAGCAAGACGTTTCCCGTTGAATATGGCTCATAACACCCCTTGTATTACTGTTTATGTAAGCAGACAGTTTTATTGTTCATGACCAAAATCCCTTAACGTGAGTTTTCGTTCCACTGAGCGTCAGACCCCGTAGAAAAGATCAAAGGATCTTCTTGAGATCCTTTTTTTCTGCGCGTAATCTGCTGCTTGCAAACAAAAAAACCACCGCTACCAGCGGTGGTTTGTTTGCCGGATCAAGAGCTACCAACTCTTTTTCCGAAGGTAACTGGCTTCAGCAGAGCGCAGATACCAAATACTGTCCTTCTAGTGTAGCCGTAGTTAGGCCACCACTTCAAGAACTCTGTAGCACCGCCTACATACCTCGCTCTGCTAATCCTGTTACCAGTGGCTGCTGCCAGTGGCGATAAGTCGTGTCTTACCGGGTTGGACTCAAGACGATAGTTACCGGATAAGGCGCAGCGGTCGGGCTGAACGGGGGGTTCGTGCACACAGCCCAGCTTGGAGCGAACGACCTACACCGAACTGAGATACCTACAGCGTGAGCTATGAGAAAGCGCCACGCTTCCCGAAGGGAGAAAGGCGGACAGGTATCCGGTAAGCGGCAGGGTCGGAACAGGAGAGCGCACGAGGGAGCTTCCAGGGGGAAACGCCTGGTATCTTTATAGTCCTGTCGGGTTTCGCCACCTCTGACTTGAGCGTCGATTTTTGTGATGCTCGTCAGGGGGGCGGAGCCTATGGAAAAACGCCAGCAACGCGGCCTTTTTACGGTTCCTGGCCTTTTGCTGGCCTTTTGCTCACATGTTCTTTCCTGCGTTATCCCCTGATTCTGTGGATAACCGTATTACCGCCTTTGAGTGAGCTGATACCGCTCGCCGCAGCCGAACGACCGAGCGCAGCGAGTCAGTGAGCGAGGAAGCGGAAGAGCGCCTGATGCGGTATTTTCTCCTTACGCATCTGTGCGGTATTTCACACCGCATATATGGTGCACTCTCAGTACAATCTGCTCTGATGCCGCATAGTTAAGCCAGTATACACTCCGCTATCGCTACGTGACTGGGTCATGGCTGCGCCCCGACACCCGCCAACACCCGCTGACGCGCCCTGACGGGCTTGTCTGCTCCCGGCATCCGCTTACAGACAAGCTGTGACCGTCTCCGGGAGCTGCATGTGTCAGAGGTTTTCACCGTCATCACCGAAACGCGCGAGGCAGCTGCGGTAAAGCTCATCAGCGTGGTCGTGAAGCGATTCACAGATGTCTGCCTGTTCATCCGCGTCCAGCTCGTTGAGTTTCTCCAGAAGCGTTAATGTCTGGCTTCTGATAAAGCGGGCCATGTTAAGGGCGGTTTTTTCCTGTTTGGTCACTGATGCCTCCGTGTAAGGGGGATTTCTGTTCATGGGGGTAATGATACCGATGAAACGAGAGAGGATGCTCACGATACGGGTTACTGATGATGAACATGCCCGGTTACTGGAACGTTGTGAGGGTAAACAACTGGCGGTATGGATGCGGCGGGACCAGAGAAAAATCACTCAGGGTCAATGCCAGCGCTTCGTTAATACAGATGTAGGTGTTCCACAGGGTAGCCAGCAGCATCCTGCGATGCAGATCCGGAACATAATGGTGCAGGGCGCTGACTTCCGCGTTTCCAGACTTTACGAAACACGGAAACCGAAGACCATTCATGTTGTTGCTCAGGTCGCAGACGTTTTGCAGCAGCAGTCGCTTCACGTTCGCTCGCGTATCGGTGATTCATTCTGCTAACCAGTAAGGCAACCCCGCCAGCCTAGCCGGGTCCTCAACGACAGGAGCACGATCATGCGCACCCGTGGGGCCGCCATGCCGGCGATAATGGCCTGCTTCTCGCCGAAACGTTTGGTGGCGGGACCAGTGACGAAGGCTTGAGCGAGGGCGTGCAAGATTCCGAATACCGCAAGCGACAGGCCGATCATCGTCGCGCTCCAGCGAAAGCGGTCCTCGCCGAAAATGACCCAGAGCGCTGCCGGCACCTGTCCTACGAGTTGCATGATAAAGAAGACAGTCATAAGTGCGGCGACGATAGTCATGCCCCGCGCCCACCGGAAGGAGCTGACTGGGTTGAAGGCTCTCAAGGGCATCGGTCGAGATCCCGGTGCCTAATGAGTGAGCTAACTTACATTAATTGCGTTGCGCTCACTGCCCGCTTTCCAGTCGGGAAACCTGTCGTGCCAGCTGCATTAATGAATCGGCCAACGCGCGGGGAGAGGCGGTTTGCGTATTGGGCGCCAGGGTGGTTTTTCTTTTCACCAGTGAGACGGGCAACAGCTGATTGCCCTTCACCGCCTGGCCCTGAGAGAGTTGCAGCAAGCGGTCCACGCTGGTTTGCCCCAGCAGGCGAAAATCCTGTTTGATGGTGGTTAACGGCGGGATATAACATGAGCTGTCTTCGGTATCGTCGTATCCCACTACCGAGATATCCGCACCAACGCGCAGCCCGGACTCGGTAATGGCGCGCATTGCGCCCAGCGCCATCTGATCGTTGGCAACCAGCATCGCAGTGGGAACGATGCCCTCATTCAGCATTTGCATGGTTTGTTGAAAACCGGACATGGCACTCCAGTCGCCTTCCCGTTCCGCTATCGGCTGAATTTGATTGCGAGTGAGATATTTATGCCAGCCAGCCAGACGCAGACGCGCCGAGACAGAACTTAATGGGCCCGCTAACAGCGCGATTTGCTGGTGACCCAATGCGACCAGATGCTCCACGCCCAGTCGCGTACCGTCTTCATGGGAGAAAATAATACTGTTGATGGGTGTCTGGTCAGAGACATCAAGAAATAACGCCGGAACATTAGTGCAGGCAGCTTCCACAGCAATGGCATCCTGGTCATCCAGCGGATAGTTAATGATCAGCCCACTGACGCGTTGCGCGAGAAGATTGTGCACCGCCGCTTTACAGGCTTCGACGCCGCTTCGTTCTACCATCGACACCACCACGCTGGCACCCAGTTGATCGGCGCGAGATTTAATCGCCGCGACAATTTGCGACGGCGCGTGCAGGGCCAGACTGGAGGTGGCAACGCCAATCAGCAACGACTGTTTGCCCGCCAGTTGTTGTGCCACGCGGTTGGGAATGTAATTCAGCTCCGCCATCGCCGCTTCCACTTTTTCCCGCGTTTTCGCAGAAACGTGGCTGGCCTGGTTCACCACGCGGGAAACGGTCTGATAAGAGACACCGGCATACTCTGCGACATCGTATAACGTTACTGGTTTCACATTCACCACCCTGAATTGACTCTCTTCCGGGCGCTATCATGCCATACCGCGAAAGGTTTTGCGCCATTCGATGGTGTCCGGGATCTCGACGCTCTCCCTTATGCGACTCCTGCATTAGGAAGCAGCCCAGTAGTAGGTTGAGGCCGTTGAGCACCGCCGCCGCAAGGAATGGTGCATGCAAGGAGATGGCGCCCAACAGTCCCCCGGCCACGGGGCCTGCCACCATACCCACGCCGAAACAAGCGCTCATGAGCCCGAAGTGGCGAGCCCGATCTTCCCCATCGGTGATGTCGGCGATATAGGCGCCAGCAACCGCACCTGTGGCGCCGGTGATGCCGGCCACGATGCGTCCGGCGTAGAGGATCGAGATCTCGATCCCGCGAAATTAATACGACTCACTATAGGGGAATTGTGAGCGGATAACAATTCCCCTCTAGAAATAATTTTGTTTAACTTTAAGAAGGAGATATACCATGAAATACCTATTGCCTACGGCAGCCGCTGGATTGTTATTACTCGCGGCCCAGCCGGCCATGGCATCTAGGACTCCGGAAATGCCGGTTCTGGAAAATCGTGCAGCACAGGGTGATATTACCGCACCGGGTGGTGCACGTCGTCTGACCGGTGATCAGACCGCAGCACTGCGTGATAGCCTGAGCGATAAACCGGCAAAAAACATTATTCTGCTGATTGGTGATGGCATGGGTGATAGCGAAATTACCGCAGCACGTAATTATGCCGAAGGTGCCGGTGGTTTTTTTAAAGGTATTGATGCACTGCCGCTGACAGGTCAGTATACCCATTATGCACTGAATAAAAAAACCGGCAAACCGGATTATGTTACCGATAGCGCAGCAAGCGCAACCGCATGGTCAACCGGTGTTAAAACCTATAATGGTGCACTGGGTGTGGATATTCATGAAAAAGATCATCCGACCATTCTGGAAATGGCAAAAGCAGCAGGTCTGGCAACCGGTAATGTTAGCACCGCAGAACTGCAGGATGCAACACCGGCAGCACTGGTTGCACATGTTACCAGCCGTAAATGTTATGGTCCGAGCGCAACCAGCGAAAAATGTCCGGGTAATGCACTGGAAAAAGGTGGTAAAGGTAGCATTACCGAACAGCTGCTGAATGCACGTGCAGATGTTACCCTGGGTGGTGGTGCAAAAACCTTTGCAGAAACCGCAACCGCAGGCGAATGGCAGGGTAAAACCCTGCGTGAACAGGCACAGGCACGTGGTTATCAGCTGGTTAGTGATGCAGCAAGCCTGAATAGCGTTACCGAAGCAAATCAGCAGAAACCGCTGCTGGGTCTGTTTGCAGATGGTAATATGCCGGTTCGTTGGCTGGGTCCGAAAGCAACCTATCATGGTAATATTGATAAACCGGCTGTTACCTGTACCCCGAATCCGCAGCGTAATGATAGCGTTCCGACCCTGGCACAGATGACCGATAAAGCAATTGAACTGCTGAGCAAAAATGAAAAAGGCTTTTTTCTGCAGGTTGAAGGTGCCAGCATTGATAAACAGGATCATGCAGCAAATCCGTGTGGTCAGATTGGTGAAACCGTTGATCTGGATGAAGCAGTTCAGCGTGCACTGGAATTTGCAAAAAAAGAAGGTAATACCCTGGTTATTGTGACCGCAGATCATGCACATGCAAGCCAGATTGTTGCACCGGATACCAAAGCACCGGGTCTGACCCAGGCACTGAATACCAAAGATGGTGCAGTTATGGTTATGAGCTATGGTAATAGCGAAGAAGATAGCCAGGAACATACCGGTAGCCAGCTGCGTATTGCAGCATATGGTCCGCATGCAGCCAATGTTGTTGGTCTGACCGATCAAACCGACCTGTTTTATACCATGAAAGCAGCACTGGGTCTGAAAGGCGGCGGTGGTAGCAAAGGTGGTGGCTCTGCAGGTGAGGCTTTAGCGTGGGGTCAGTGTGGGGGTAGACGTCCCACCGATACACGCCCTTGCGCTGATGGAGCCCTTTGCATAGTCCGGAATGAACGCACCAATTTCTCTCAGTGCGTTCCGTCCGGAGGTTCAAAGGGCGAGCACCACCACCACCACCACTGAGATCCGGCTGCTAACAAAGCCCGAAAGGAAGCTGAGTTGGCTGCTGCCACCGCTGAGCAATAACTAGCATAACCCCTTGGGGCCTCTAAACGGGTCTTGAGGGGTTTTTTGCTGAAAGGAGGAACTATATCCGGAT

**pBR3k pelB-AP-EHUX4**

TGGCGAATGGGACGCGCCCTGTAGCGGCGCATTAAGCGCGGCGGGTGTGGTGGTTACGCGCAGCGTGACCGCTACACTTGCCAGCGCCCTAGCGCCCGCTCCTTTCGCTTTCTTCCCTTCCTTTCTCGCCACGTTCGCCGGCTTTCCCCGTCAAGCTCTAAATCGGGGGCTCCCTTTAGGGTTCCGATTTAGTGCTTTACGGCACCTCGACCCCAAAAAACTTGATTAGGGTGATGGTTCACGTAGTGGGCCATCGCCCTGATAGACGGTTTTTCGCCCTTTGACGTTGGAGTCCACGTTCTTTAATAGTGGACTCTTGTTCCAAACTGGAACAACACTCAACCCTATCTCGGTCTATTCTTTTGATTTATAAGGGATTTTGCCGATTTCGGCCTATTGGTTAAAAAATGAGCTGATTTAACAAAAATTTAACGCGAATTTTAACAAAATATTAACGCTTACAATTTAGGTGGCACTTTTCGGGGAAATGTGCGCGGAACCCCTATTTGTTTATTTTTCTAAATACATTCAAATATGTATCCGCTCATGAATTAATTCTTAGAAAAACTCATCGAGCATCAAATGAAACTGCAATTTATTCATATCAGGATTATCAATACCATATTTTTGAAAAAGCCGTTTCTGTAATGAAGGAGAAAACTCACCGAGGCAGTTCCATAGGATGGCAAGATCCTGGTATCGGTCTGCGATTCCGACTCGTCCAACATCAATACAACCTATTAATTTCCCCTCGTCAAAAATAAGGTTATCAAGTGAGAAATCACCATGAGTGACGACTGAATCCGGTGAGAATGGCAAAAGTTTATGCATTTCTTTCCAGACTTGTTCAACAGGCCAGCCATTACGCTCGTCATCAAAATCACTCGCATCAACCAAACCGTTATTCATTCGTGATTGCGCCTGAGCGAGACGAAATACGCGATCGCTGTTAAAAGGACAATTACAAACAGGAATCGAATGCAACCGGCGCAGGAACACTGCCAGCGCATCAACAATATTTTCACCTGAATCAGGATATTCTTCTAATACCTGGAATGCTGTTTTCCCGGGGATCGCAGTGGTGAGTAACCATGCATCATCAGGAGTACGGATAAAATGCTTGATGGTCGGAAGAGGCATAAATTCCGTCAGCCAGTTTAGTCTGACCATCTCATCTGTAACATCATTGGCAACGCTACCTTTGCCATGTTTCAGAAACAACTCTGGCGCATCGGGCTTCCCATACAATCGATAGATTGTCGCACCTGATTGCCCGACATTATCGCGAGCCCATTTATACCCATATAAATCAGCATCCATGTTGGAATTTAATCGCGGCCTAGAGCAAGACGTTTCCCGTTGAATATGGCTCATAACACCCCTTGTATTACTGTTTATGTAAGCAGACAGTTTTATTGTTCATGACCAAAATCCCTTAACGTGAGTTTTCGTTCCACTGAGCGTCAGACCCCGTAGAAAAGATCAAAGGATCTTCTTGAGATCCTTTTTTTCTGCGCGTAATCTGCTGCTTGCAAACAAAAAAACCACCGCTACCAGCGGTGGTTTGTTTGCCGGATCAAGAGCTACCAACTCTTTTTCCGAAGGTAACTGGCTTCAGCAGAGCGCAGATACCAAATACTGTCCTTCTAGTGTAGCCGTAGTTAGGCCACCACTTCAAGAACTCTGTAGCACCGCCTACATACCTCGCTCTGCTAATCCTGTTACCAGTGGCTGCTGCCAGTGGCGATAAGTCGTGTCTTACCGGGTTGGACTCAAGACGATAGTTACCGGATAAGGCGCAGCGGTCGGGCTGAACGGGGGGTTCGTGCACACAGCCCAGCTTGGAGCGAACGACCTACACCGAACTGAGATACCTACAGCGTGAGCTATGAGAAAGCGCCACGCTTCCCGAAGGGAGAAAGGCGGACAGGTATCCGGTAAGCGGCAGGGTCGGAACAGGAGAGCGCACGAGGGAGCTTCCAGGGGGAAACGCCTGGTATCTTTATAGTCCTGTCGGGTTTCGCCACCTCTGACTTGAGCGTCGATTTTTGTGATGCTCGTCAGGGGGGCGGAGCCTATGGAAAAACGCCAGCAACGCGGCCTTTTTACGGTTCCTGGCCTTTTGCTGGCCTTTTGCTCACATGTTCTTTCCTGCGTTATCCCCTGATTCTGTGGATAACCGTATTACCGCCTTTGAGTGAGCTGATACCGCTCGCCGCAGCCGAACGACCGAGCGCAGCGAGTCAGTGAGCGAGGAAGCGGAAGAGCGCCTGATGCGGTATTTTCTCCTTACGCATCTGTGCGGTATTTCACACCGCATATATGGTGCACTCTCAGTACAATCTGCTCTGATGCCGCATAGTTAAGCCAGTATACACTCCGCTATCGCTACGTGACTGGGTCATGGCTGCGCCCCGACACCCGCCAACACCCGCTGACGCGCCCTGACGGGCTTGTCTGCTCCCGGCATCCGCTTACAGACAAGCTGTGACCGTCTCCGGGAGCTGCATGTGTCAGAGGTTTTCACCGTCATCACCGAAACGCGCGAGGCAGCTGCGGTAAAGCTCATCAGCGTGGTCGTGAAGCGATTCACAGATGTCTGCCTGTTCATCCGCGTCCAGCTCGTTGAGTTTCTCCAGAAGCGTTAATGTCTGGCTTCTGATAAAGCGGGCCATGTTAAGGGCGGTTTTTTCCTGTTTGGTCACTGATGCCTCCGTGTAAGGGGGATTTCTGTTCATGGGGGTAATGATACCGATGAAACGAGAGAGGATGCTCACGATACGGGTTACTGATGATGAACATGCCCGGTTACTGGAACGTTGTGAGGGTAAACAACTGGCGGTATGGATGCGGCGGGACCAGAGAAAAATCACTCAGGGTCAATGCCAGCGCTTCGTTAATACAGATGTAGGTGTTCCACAGGGTAGCCAGCAGCATCCTGCGATGCAGATCCGGAACATAATGGTGCAGGGCGCTGACTTCCGCGTTTCCAGACTTTACGAAACACGGAAACCGAAGACCATTCATGTTGTTGCTCAGGTCGCAGACGTTTTGCAGCAGCAGTCGCTTCACGTTCGCTCGCGTATCGGTGATTCATTCTGCTAACCAGTAAGGCAACCCCGCCAGCCTAGCCGGGTCCTCAACGACAGGAGCACGATCATGCGCACCCGTGGGGCCGCCATGCCGGCGATAATGGCCTGCTTCTCGCCGAAACGTTTGGTGGCGGGACCAGTGACGAAGGCTTGAGCGAGGGCGTGCAAGATTCCGAATACCGCAAGCGACAGGCCGATCATCGTCGCGCTCCAGCGAAAGCGGTCCTCGCCGAAAATGACCCAGAGCGCTGCCGGCACCTGTCCTACGAGTTGCATGATAAAGAAGACAGTCATAAGTGCGGCGACGATAGTCATGCCCCGCGCCCACCGGAAGGAGCTGACTGGGTTGAAGGCTCTCAAGGGCATCGGTCGAGATCCCGGTGCCTAATGAGTGAGCTAACTTACATTAATTGCGTTGCGCTCACTGCCCGCTTTCCAGTCGGGAAACCTGTCGTGCCAGCTGCATTAATGAATCGGCCAACGCGCGGGGAGAGGCGGTTTGCGTATTGGGCGCCAGGGTGGTTTTTCTTTTCACCAGTGAGACGGGCAACAGCTGATTGCCCTTCACCGCCTGGCCCTGAGAGAGTTGCAGCAAGCGGTCCACGCTGGTTTGCCCCAGCAGGCGAAAATCCTGTTTGATGGTGGTTAACGGCGGGATATAACATGAGCTGTCTTCGGTATCGTCGTATCCCACTACCGAGATATCCGCACCAACGCGCAGCCCGGACTCGGTAATGGCGCGCATTGCGCCCAGCGCCATCTGATCGTTGGCAACCAGCATCGCAGTGGGAACGATGCCCTCATTCAGCATTTGCATGGTTTGTTGAAAACCGGACATGGCACTCCAGTCGCCTTCCCGTTCCGCTATCGGCTGAATTTGATTGCGAGTGAGATATTTATGCCAGCCAGCCAGACGCAGACGCGCCGAGACAGAACTTAATGGGCCCGCTAACAGCGCGATTTGCTGGTGACCCAATGCGACCAGATGCTCCACGCCCAGTCGCGTACCGTCTTCATGGGAGAAAATAATACTGTTGATGGGTGTCTGGTCAGAGACATCAAGAAATAACGCCGGAACATTAGTGCAGGCAGCTTCCACAGCAATGGCATCCTGGTCATCCAGCGGATAGTTAATGATCAGCCCACTGACGCGTTGCGCGAGAAGATTGTGCACCGCCGCTTTACAGGCTTCGACGCCGCTTCGTTCTACCATCGACACCACCACGCTGGCACCCAGTTGATCGGCGCGAGATTTAATCGCCGCGACAATTTGCGACGGCGCGTGCAGGGCCAGACTGGAGGTGGCAACGCCAATCAGCAACGACTGTTTGCCCGCCAGTTGTTGTGCCACGCGGTTGGGAATGTAATTCAGCTCCGCCATCGCCGCTTCCACTTTTTCCCGCGTTTTCGCAGAAACGTGGCTGGCCTGGTTCACCACGCGGGAAACGGTCTGATAAGAGACACCGGCATACTCTGCGACATCGTATAACGTTACTGGTTTCACATTCACCACCCTGAATTGACTCTCTTCCGGGCGCTATCATGCCATACCGCGAAAGGTTTTGCGCCATTCGATGGTGTCCGGGATCTCGACGCTCTCCCTTATGCGACTCCTGCATTAGGAAGCAGCCCAGTAGTAGGTTGAGGCCGTTGAGCACCGCCGCCGCAAGGAATGGTGCATGCAAGGAGATGGCGCCCAACAGTCCCCCGGCCACGGGGCCTGCCACCATACCCACGCCGAAACAAGCGCTCATGAGCCCGAAGTGGCGAGCCCGATCTTCCCCATCGGTGATGTCGGCGATATAGGCGCCAGCAACCGCACCTGTGGCGCCGGTGATGCCGGCCACGATGCGTCCGGCGTAGAGGATCGAGATCTCGATCCCGCGAAATTAATACGACTCACTATAGGGGAATTGTGAGCGGATAACAATTCCCCTCTAGAAATAATTTTGTTTAACTTTAAGAAGGAGATATACCATGAAATACCTATTGCCTACGGCAGCCGCTGGATTGTTATTACTCGCGGCCCAGCCGGCCATGGCATCTAGGACTCCGGAAATGCCGGTTCTGGAAAATCGTGCAGCACAGGGTGATATTACCGCACCGGGTGGTGCACGTCGTCTGACCGGTGATCAGACCGCAGCACTGCGTGATAGCCTGAGCGATAAACCGGCAAAAAACATTATTCTGCTGATTGGTGATGGCATGGGTGATAGCGAAATTACCGCAGCACGTAATTATGCCGAAGGTGCCGGTGGTTTTTTTAAAGGTATTGATGCACTGCCGCTGACAGGTCAGTATACCCATTATGCACTGAATAAAAAAACCGGCAAACCGGATTATGTTACCGATAGCGCAGCAAGCGCAACCGCATGGTCAACCGGTGTTAAAACCTATAATGGTGCACTGGGTGTGGATATTCATGAAAAAGATCATCCGACCATTCTGGAAATGGCAAAAGCAGCAGGTCTGGCAACCGGTAATGTTAGCACCGCAGAACTGCAGGATGCAACACCGGCAGCACTGGTTGCACATGTTACCAGCCGTAAATGTTATGGTCCGAGCGCAACCAGCGAAAAATGTCCGGGTAATGCACTGGAAAAAGGTGGTAAAGGTAGCATTACCGAACAGCTGCTGAATGCACGTGCAGATGTTACCCTGGGTGGTGGTGCAAAAACCTTTGCAGAAACCGCAACCGCAGGCGAATGGCAGGGTAAAACCCTGCGTGAACAGGCACAGGCACGTGGTTATCAGCTGGTTAGTGATGCAGCAAGCCTGAATAGCGTTACCGAAGCAAATCAGCAGAAACCGCTGCTGGGTCTGTTTGCAGATGGTAATATGCCGGTTCGTTGGCTGGGTCCGAAAGCAACCTATCATGGTAATATTGATAAACCGGCTGTTACCTGTACCCCGAATCCGCAGCGTAATGATAGCGTTCCGACCCTGGCACAGATGACCGATAAAGCAATTGAACTGCTGAGCAAAAATGAAAAAGGCTTTTTTCTGCAGGTTGAAGGTGCCAGCATTGATAAACAGGATCATGCAGCAAATCCGTGTGGTCAGATTGGTGAAACCGTTGATCTGGATGAAGCAGTTCAGCGTGCACTGGAATTTGCAAAAAAAGAAGGTAATACCCTGGTTATTGTGACCGCAGATCATGCACATGCAAGCCAGATTGTTGCACCGGATACCAAAGCACCGGGTCTGACCCAGGCACTGAATACCAAAGATGGTGCAGTTATGGTTATGAGCTATGGTAATAGCGAAGAAGATAGCCAGGAACATACCGGTAGCCAGCTGCGTATTGCAGCATATGGTCCGCATGCAGCCAATGTTGTTGGTCTGACCGATCAAACCGACCTGTTTTATACCATGAAAGCAGCACTGGGTCTGAAAGGCGGCGGTGGTAGCAAAGGTGGTGGCTCTACGATCTGTCAGCTGAAGTGGGCACAGTGCGGCGGAAATTCCTGGACTGGGGCCACCTGTTGTGAGGCTGGTTCAGTGTGCACCGCCTCTTCTGCATGGTATTCTCAATGTAAACCCAGCCGGAATTATCTGGGAGGTTCAAAGGGCGAGCACCACCACCACCACCACTGAGATCCGGCTGCTAACAAAGCCCGAAAGGAAGCTGAGTTGGCTGCTGCCACCGCTGAGCAATAACTAGCATAACCCCTTGGGGCCTCTAAACGGGTCTTGAGGGGTTTTTTGCTGAAAGGAGGAACTATATCCGGAT

**pBR3l pelB-AP-EHUX5**

TGGCGAATGGGACGCGCCCTGTAGCGGCGCATTAAGCGCGGCGGGTGTGGTGGTTACGCGCAGCGTGACCGCTACACTTGCCAGCGCCCTAGCGCCCGCTCCTTTCGCTTTCTTCCCTTCCTTTCTCGCCACGTTCGCCGGCTTTCCCCGTCAAGCTCTAAATCGGGGGCTCCCTTTAGGGTTCCGATTTAGTGCTTTACGGCACCTCGACCCCAAAAAACTTGATTAGGGTGATGGTTCACGTAGTGGGCCATCGCCCTGATAGACGGTTTTTCGCCCTTTGACGTTGGAGTCCACGTTCTTTAATAGTGGACTCTTGTTCCAAACTGGAACAACACTCAACCCTATCTCGGTCTATTCTTTTGATTTATAAGGGATTTTGCCGATTTCGGCCTATTGGTTAAAAAATGAGCTGATTTAACAAAAATTTAACGCGAATTTTAACAAAATATTAACGCTTACAATTTAGGTGGCACTTTTCGGGGAAATGTGCGCGGAACCCCTATTTGTTTATTTTTCTAAATACATTCAAATATGTATCCGCTCATGAATTAATTCTTAGAAAAACTCATCGAGCATCAAATGAAACTGCAATTTATTCATATCAGGATTATCAATACCATATTTTTGAAAAAGCCGTTTCTGTAATGAAGGAGAAAACTCACCGAGGCAGTTCCATAGGATGGCAAGATCCTGGTATCGGTCTGCGATTCCGACTCGTCCAACATCAATACAACCTATTAATTTCCCCTCGTCAAAAATAAGGTTATCAAGTGAGAAATCACCATGAGTGACGACTGAATCCGGTGAGAATGGCAAAAGTTTATGCATTTCTTTCCAGACTTGTTCAACAGGCCAGCCATTACGCTCGTCATCAAAATCACTCGCATCAACCAAACCGTTATTCATTCGTGATTGCGCCTGAGCGAGACGAAATACGCGATCGCTGTTAAAAGGACAATTACAAACAGGAATCGAATGCAACCGGCGCAGGAACACTGCCAGCGCATCAACAATATTTTCACCTGAATCAGGATATTCTTCTAATACCTGGAATGCTGTTTTCCCGGGGATCGCAGTGGTGAGTAACCATGCATCATCAGGAGTACGGATAAAATGCTTGATGGTCGGAAGAGGCATAAATTCCGTCAGCCAGTTTAGTCTGACCATCTCATCTGTAACATCATTGGCAACGCTACCTTTGCCATGTTTCAGAAACAACTCTGGCGCATCGGGCTTCCCATACAATCGATAGATTGTCGCACCTGATTGCCCGACATTATCGCGAGCCCATTTATACCCATATAAATCAGCATCCATGTTGGAATTTAATCGCGGCCTAGAGCAAGACGTTTCCCGTTGAATATGGCTCATAACACCCCTTGTATTACTGTTTATGTAAGCAGACAGTTTTATTGTTCATGACCAAAATCCCTTAACGTGAGTTTTCGTTCCACTGAGCGTCAGACCCCGTAGAAAAGATCAAAGGATCTTCTTGAGATCCTTTTTTTCTGCGCGTAATCTGCTGCTTGCAAACAAAAAAACCACCGCTACCAGCGGTGGTTTGTTTGCCGGATCAAGAGCTACCAACTCTTTTTCCGAAGGTAACTGGCTTCAGCAGAGCGCAGATACCAAATACTGTCCTTCTAGTGTAGCCGTAGTTAGGCCACCACTTCAAGAACTCTGTAGCACCGCCTACATACCTCGCTCTGCTAATCCTGTTACCAGTGGCTGCTGCCAGTGGCGATAAGTCGTGTCTTACCGGGTTGGACTCAAGACGATAGTTACCGGATAAGGCGCAGCGGTCGGGCTGAACGGGGGGTTCGTGCACACAGCCCAGCTTGGAGCGAACGACCTACACCGAACTGAGATACCTACAGCGTGAGCTATGAGAAAGCGCCACGCTTCCCGAAGGGAGAAAGGCGGACAGGTATCCGGTAAGCGGCAGGGTCGGAACAGGAGAGCGCACGAGGGAGCTTCCAGGGGGAAACGCCTGGTATCTTTATAGTCCTGTCGGGTTTCGCCACCTCTGACTTGAGCGTCGATTTTTGTGATGCTCGTCAGGGGGGCGGAGCCTATGGAAAAACGCCAGCAACGCGGCCTTTTTACGGTTCCTGGCCTTTTGCTGGCCTTTTGCTCACATGTTCTTTCCTGCGTTATCCCCTGATTCTGTGGATAACCGTATTACCGCCTTTGAGTGAGCTGATACCGCTCGCCGCAGCCGAACGACCGAGCGCAGCGAGTCAGTGAGCGAGGAAGCGGAAGAGCGCCTGATGCGGTATTTTCTCCTTACGCATCTGTGCGGTATTTCACACCGCATATATGGTGCACTCTCAGTACAATCTGCTCTGATGCCGCATAGTTAAGCCAGTATACACTCCGCTATCGCTACGTGACTGGGTCATGGCTGCGCCCCGACACCCGCCAACACCCGCTGACGCGCCCTGACGGGCTTGTCTGCTCCCGGCATCCGCTTACAGACAAGCTGTGACCGTCTCCGGGAGCTGCATGTGTCAGAGGTTTTCACCGTCATCACCGAAACGCGCGAGGCAGCTGCGGTAAAGCTCATCAGCGTGGTCGTGAAGCGATTCACAGATGTCTGCCTGTTCATCCGCGTCCAGCTCGTTGAGTTTCTCCAGAAGCGTTAATGTCTGGCTTCTGATAAAGCGGGCCATGTTAAGGGCGGTTTTTTCCTGTTTGGTCACTGATGCCTCCGTGTAAGGGGGATTTCTGTTCATGGGGGTAATGATACCGATGAAACGAGAGAGGATGCTCACGATACGGGTTACTGATGATGAACATGCCCGGTTACTGGAACGTTGTGAGGGTAAACAACTGGCGGTATGGATGCGGCGGGACCAGAGAAAAATCACTCAGGGTCAATGCCAGCGCTTCGTTAATACAGATGTAGGTGTTCCACAGGGTAGCCAGCAGCATCCTGCGATGCAGATCCGGAACATAATGGTGCAGGGCGCTGACTTCCGCGTTTCCAGACTTTACGAAACACGGAAACCGAAGACCATTCATGTTGTTGCTCAGGTCGCAGACGTTTTGCAGCAGCAGTCGCTTCACGTTCGCTCGCGTATCGGTGATTCATTCTGCTAACCAGTAAGGCAACCCCGCCAGCCTAGCCGGGTCCTCAACGACAGGAGCACGATCATGCGCACCCGTGGGGCCGCCATGCCGGCGATAATGGCCTGCTTCTCGCCGAAACGTTTGGTGGCGGGACCAGTGACGAAGGCTTGAGCGAGGGCGTGCAAGATTCCGAATACCGCAAGCGACAGGCCGATCATCGTCGCGCTCCAGCGAAAGCGGTCCTCGCCGAAAATGACCCAGAGCGCTGCCGGCACCTGTCCTACGAGTTGCATGATAAAGAAGACAGTCATAAGTGCGGCGACGATAGTCATGCCCCGCGCCCACCGGAAGGAGCTGACTGGGTTGAAGGCTCTCAAGGGCATCGGTCGAGATCCCGGTGCCTAATGAGTGAGCTAACTTACATTAATTGCGTTGCGCTCACTGCCCGCTTTCCAGTCGGGAAACCTGTCGTGCCAGCTGCATTAATGAATCGGCCAACGCGCGGGGAGAGGCGGTTTGCGTATTGGGCGCCAGGGTGGTTTTTCTTTTCACCAGTGAGACGGGCAACAGCTGATTGCCCTTCACCGCCTGGCCCTGAGAGAGTTGCAGCAAGCGGTCCACGCTGGTTTGCCCCAGCAGGCGAAAATCCTGTTTGATGGTGGTTAACGGCGGGATATAACATGAGCTGTCTTCGGTATCGTCGTATCCCACTACCGAGATATCCGCACCAACGCGCAGCCCGGACTCGGTAATGGCGCGCATTGCGCCCAGCGCCATCTGATCGTTGGCAACCAGCATCGCAGTGGGAACGATGCCCTCATTCAGCATTTGCATGGTTTGTTGAAAACCGGACATGGCACTCCAGTCGCCTTCCCGTTCCGCTATCGGCTGAATTTGATTGCGAGTGAGATATTTATGCCAGCCAGCCAGACGCAGACGCGCCGAGACAGAACTTAATGGGCCCGCTAACAGCGCGATTTGCTGGTGACCCAATGCGACCAGATGCTCCACGCCCAGTCGCGTACCGTCTTCATGGGAGAAAATAATACTGTTGATGGGTGTCTGGTCAGAGACATCAAGAAATAACGCCGGAACATTAGTGCAGGCAGCTTCCACAGCAATGGCATCCTGGTCATCCAGCGGATAGTTAATGATCAGCCCACTGACGCGTTGCGCGAGAAGATTGTGCACCGCCGCTTTACAGGCTTCGACGCCGCTTCGTTCTACCATCGACACCACCACGCTGGCACCCAGTTGATCGGCGCGAGATTTAATCGCCGCGACAATTTGCGACGGCGCGTGCAGGGCCAGACTGGAGGTGGCAACGCCAATCAGCAACGACTGTTTGCCCGCCAGTTGTTGTGCCACGCGGTTGGGAATGTAATTCAGCTCCGCCATCGCCGCTTCCACTTTTTCCCGCGTTTTCGCAGAAACGTGGCTGGCCTGGTTCACCACGCGGGAAACGGTCTGATAAGAGACACCGGCATACTCTGCGACATCGTATAACGTTACTGGTTTCACATTCACCACCCTGAATTGACTCTCTTCCGGGCGCTATCATGCCATACCGCGAAAGGTTTTGCGCCATTCGATGGTGTCCGGGATCTCGACGCTCTCCCTTATGCGACTCCTGCATTAGGAAGCAGCCCAGTAGTAGGTTGAGGCCGTTGAGCACCGCCGCCGCAAGGAATGGTGCATGCAAGGAGATGGCGCCCAACAGTCCCCCGGCCACGGGGCCTGCCACCATACCCACGCCGAAACAAGCGCTCATGAGCCCGAAGTGGCGAGCCCGATCTTCCCCATCGGTGATGTCGGCGATATAGGCGCCAGCAACCGCACCTGTGGCGCCGGTGATGCCGGCCACGATGCGTCCGGCGTAGAGGATCGAGATCTCGATCCCGCGAAATTAATACGACTCACTATAGGGGAATTGTGAGCGGATAACAATTCCCCTCTAGAAATAATTTTGTTTAACTTTAAGAAGGAGATATACCATGAAATACCTATTGCCTACGGCAGCCGCTGGATTGTTATTACTCGCGGCCCAGCCGGCCATGGCATCTAGGACTCCGGAAATGCCGGTTCTGGAAAATCGTGCAGCACAGGGTGATATTACCGCACCGGGTGGTGCACGTCGTCTGACCGGTGATCAGACCGCAGCACTGCGTGATAGCCTGAGCGATAAACCGGCAAAAAACATTATTCTGCTGATTGGTGATGGCATGGGTGATAGCGAAATTACCGCAGCACGTAATTATGCCGAAGGTGCCGGTGGTTTTTTTAAAGGTATTGATGCACTGCCGCTGACAGGTCAGTATACCCATTATGCACTGAATAAAAAAACCGGCAAACCGGATTATGTTACCGATAGCGCAGCAAGCGCAACCGCATGGTCAACCGGTGTTAAAACCTATAATGGTGCACTGGGTGTGGATATTCATGAAAAAGATCATCCGACCATTCTGGAAATGGCAAAAGCAGCAGGTCTGGCAACCGGTAATGTTAGCACCGCAGAACTGCAGGATGCAACACCGGCAGCACTGGTTGCACATGTTACCAGCCGTAAATGTTATGGTCCGAGCGCAACCAGCGAAAAATGTCCGGGTAATGCACTGGAAAAAGGTGGTAAAGGTAGCATTACCGAACAGCTGCTGAATGCACGTGCAGATGTTACCCTGGGTGGTGGTGCAAAAACCTTTGCAGAAACCGCAACCGCAGGCGAATGGCAGGGTAAAACCCTGCGTGAACAGGCACAGGCACGTGGTTATCAGCTGGTTAGTGATGCAGCAAGCCTGAATAGCGTTACCGAAGCAAATCAGCAGAAACCGCTGCTGGGTCTGTTTGCAGATGGTAATATGCCGGTTCGTTGGCTGGGTCCGAAAGCAACCTATCATGGTAATATTGATAAACCGGCTGTTACCTGTACCCCGAATCCGCAGCGTAATGATAGCGTTCCGACCCTGGCACAGATGACCGATAAAGCAATTGAACTGCTGAGCAAAAATGAAAAAGGCTTTTTTCTGCAGGTTGAAGGTGCCAGCATTGATAAACAGGATCATGCAGCAAATCCGTGTGGTCAGATTGGTGAAACCGTTGATCTGGATGAAGCAGTTCAGCGTGCACTGGAATTTGCAAAAAAAGAAGGTAATACCCTGGTTATTGTGACCGCAGATCATGCACATGCAAGCCAGATTGTTGCACCGGATACCAAAGCACCGGGTCTGACCCAGGCACTGAATACCAAAGATGGTGCAGTTATGGTTATGAGCTATGGTAATAGCGAAGAAGATAGCCAGGAACATACCGGTAGCCAGCTGCGTATTGCAGCATATGGTCCGCATGCAGCCAATGTTGTTGGTCTGACCGATCAAACCGACCTGTTTTATACCATGAAAGCAGCACTGGGTCTGAAAGGCGGCGGTGGTAGCAAAGGTGGTGGCTCTGACTATCCTGGCCTGACTTGGCATGCGCAATGTGGAGGGGTTGGTTTCTCAACCGATCCGCTGGCCTGCCAGGAAGGCCTGGGCTGCTACCAGAGAAATGAGTATTATTGGCAGTGTCTGGAAGCCCCAGGAGGTTCAAAGGGCGAGCACCACCACCACCACCACTGAGATCCGGCTGCTAACAAAGCCCGAAAGGAAGCTGAGTTGGCTGCTGCCACCGCTGAGCAATAACTAGCATAACCCCTTGGGGCCTCTAAACGGGTCTTGAGGGGTTTTTTGCTGAAAGGAGGAACTATATCCGGAT

**REFERENCES SUPPORTING INFORMATION**

1. Pettersen EF, Goddard TD, Huang CC, Couch GS, Greenblatt DM, Meng EC, et al. UCSF Chimera?A visualization system for exploratory research and analysis. J Comput Chem. 2004;25: 1605–1612. doi:10.1002/jcc.20084

2. Kraulis J, Clore GM, Nilges M, Jones TA, Pettersson G, Knowles J, et al. Determination of the three-dimensional solution structure of the C-terminal domain of cellobiohydrolase I from Trichoderma reesei. A study using nuclear magnetic resonance and hybrid distance geometry-dynamical simulated annealing. Biochemistry. 1989;28: 7241–57. Available: http://www.ncbi.nlm.nih.gov/pubmed/2554967

3. Biasini M, Bienert S, Waterhouse A, Arnold K, Studer G, Schmidt T, et al. SWISS-MODEL: modelling protein tertiary and quaternary structure using evolutionary information. Nucleic Acids Res. Oxford University Press; 2014;42: W252–W258. doi:10.1093/nar/gku340
