# Supplementary figures and images for: Fungal-type carbohydrate binding modules from the coccolithophore Emiliania huxleyi show binding affinity to cellulose and chitin
Source: PLoS One. 2018 May 21;13(5):e0197875. doi: 10.1371/journal.pone.0197875 (PMC5962083; doi:10.1371/journal.pone.0197875)

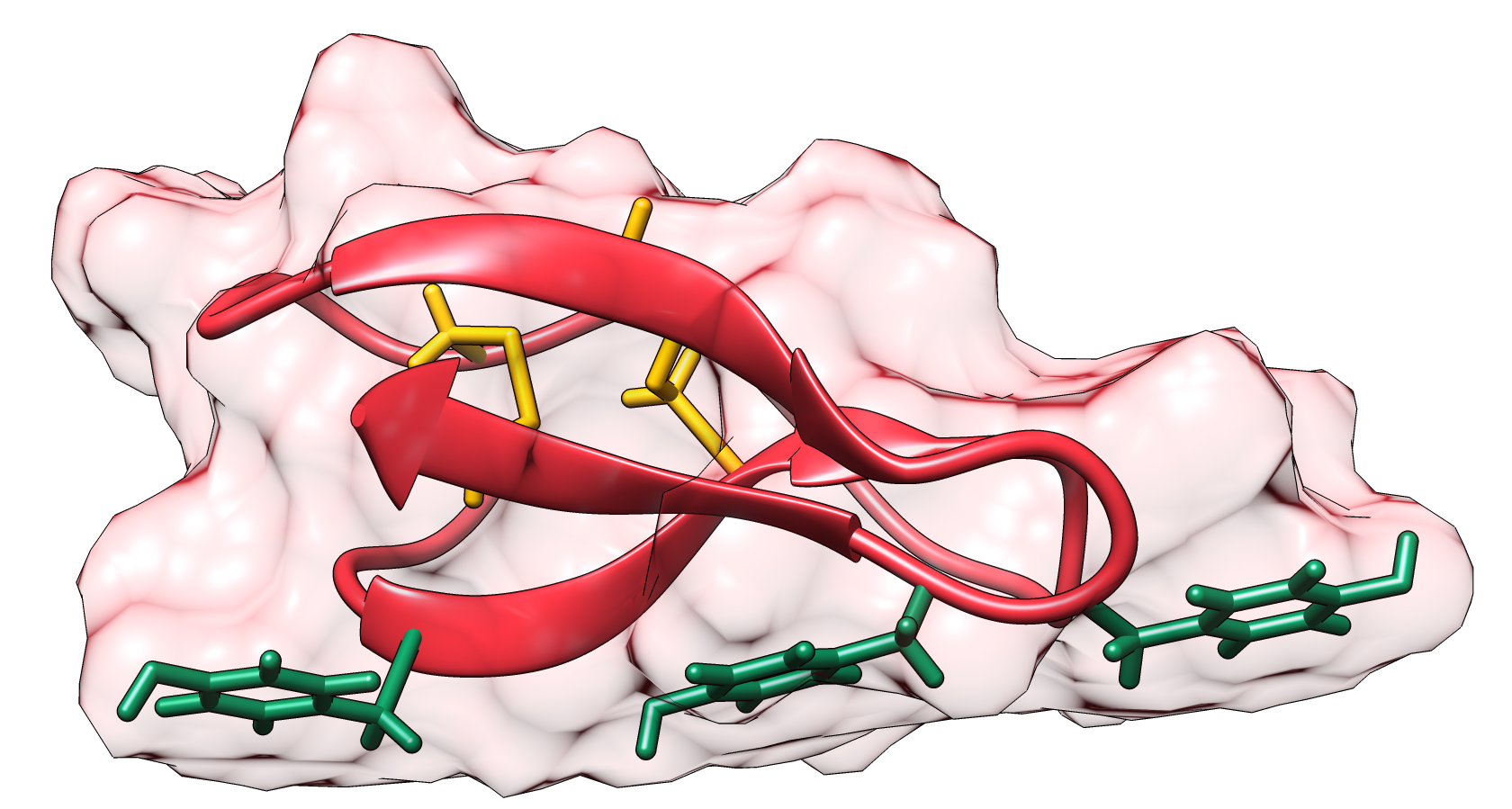

Supplement: S1 Fig — The disulfide bridges are shown in yellow, and the tyrosines involved in cellulose binding in green. Image created with Chimera, PDB ID: 1CBH. (TIF) [file pone.0197875.s002.tif]

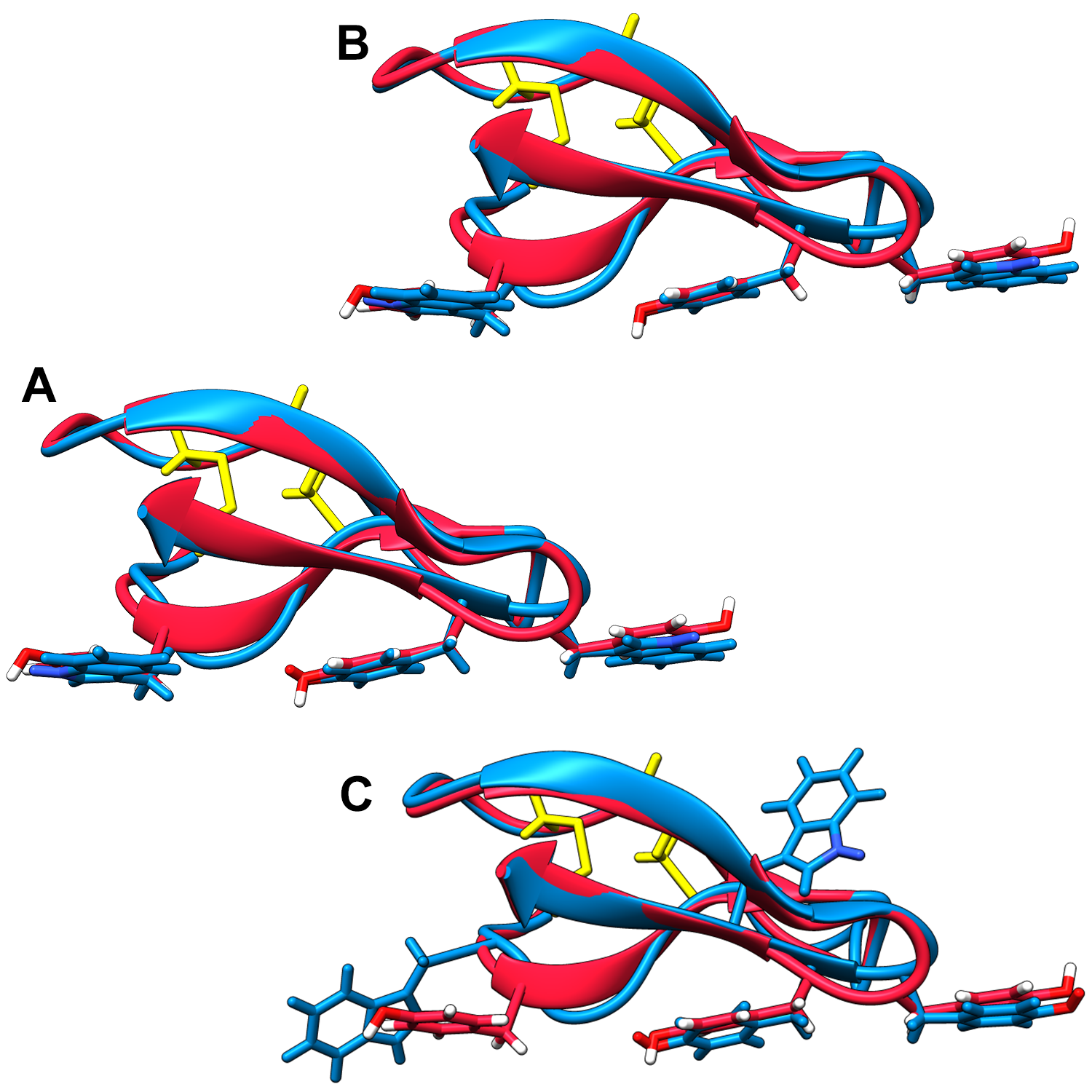

Supplement: S2 Fig — Homology models of the (A) EHUX2, (B) EHUX4 and (C) EHUX5 CBM1 domains, displayed as blue ribbons, with Cel7A-CBM1 as overlay, displayed in red. Cysteine bridges and the side-chains of residues that are important for binding are shown. Homology models were created by SWISS-MODEL with the crystal structure of Cel7A-CBM1 (PDB ID: 1CBH) as a template. (TIF) [file pone.0197875.s003.tif]

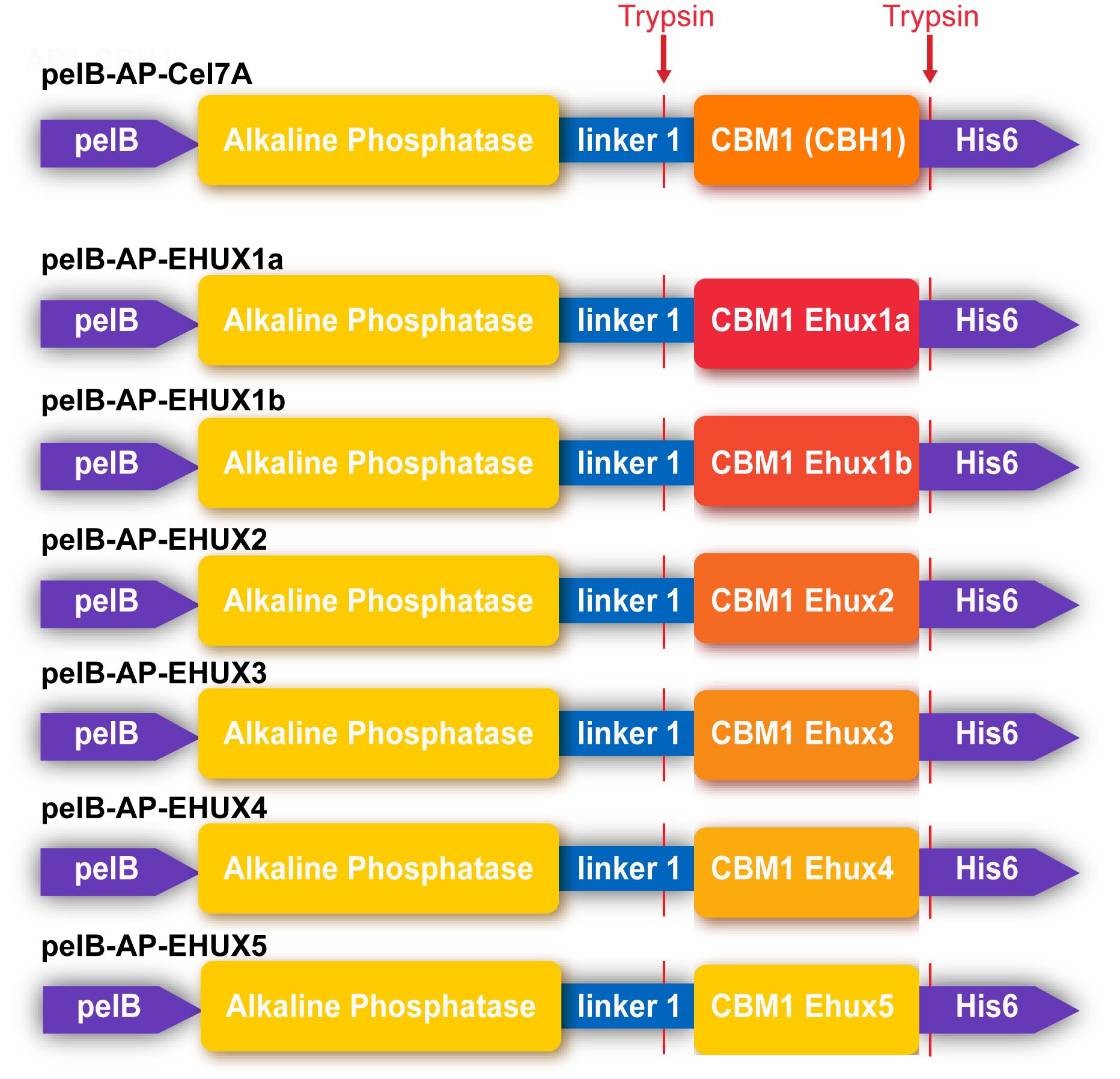

Supplement: S3 Fig — The proteins were designed to contain an N-terminal pelB signal peptide, Alkaline Phosphatase (AP) as a carrier protein and the CBM1 of interest with a His-tag on the C-terminus. The spacers flanking the CBM1 domains can be cleaved by trypsin, which does not degrade the Cel7A-CBM1 (it does degrade most of the EHUX-CBM1s). (TIF) [file pone.0197875.s004.tif]
