# Supplementary material for: Fungal-type carbohydrate binding modules from the coccolithophore Emiliania huxleyi show binding affinity to cellulose and chitin
Source: PLoS One. 2018 May 21;13(5):e0197875. doi: 10.1371/journal.pone.0197875 (PMC5962083; doi:10.1371/journal.pone.0197875)
Supplement: S1 Table — The associated functions (of the proteins, besides their CBM1 domains) were obtained with Gene Ontology (GO) and Interpro, so they are predicted based on sequence, using protein domains and families. The proteins can be found in Uniprot database using their GI. (DOCX) [file pone.0197875.s005.docx]

**Table S1**

| **Protein name** | **GI** | **Protein size (kDa)** | **Predicted associated function** |
| --- | --- | --- | --- |
| EHUX1a | R1C3S1 | 58.12 | - Transmembrane (extracellular) - hydrolase activity, hydrolyzing O-glycosyl compounds - Aspein-like, Asp-rich domain |
| EHUX1b | R1C3S1 | 58.12 | Same as EHUX1a |
| EHUX2 | R1C6A1 | 64.49 | - Transmembrane (extracellular) - hydrolase activity, hydrolyzing O-glycosyl compounds - Tyrosine Kinase activity - Amino Acid Phosphorylation |
| EHUX3 | R1EZE3 | 41.74 | - Transmembrane (extracellular) - hydrolase activity, hydrolyzing O-glycosyl compounds - Calcium Ion binding |
| EHUX4 | R1EB44 | 27.46 | - Transmembrane (extracellular) - hydrolase activity, hydrolyzing O-glycosyl compounds |
| EHUX5 | R1CQK6 | 93.42 | - Transmembrane (extracellular) - hydrolase activity, hydrolyzing O-glycosyl compounds |
